# Utilizing transcriptomics and proteomics to unravel key genes and proteins of Oryza sativa seedlings mediated by selenium in response to cadmium stress

**DOI:** 10.1186/s12870-024-05076-7

**Published:** 2024-05-03

**Authors:** Sixi Zhu, Suxia Sun, Wei Zhao, Xiuqin Yang, Huan Mao, Luying Sheng, Zhongbing Chen

**Affiliations:** 1 College of Eco-Environment Engineering, The Karst Environmental Geological Hazard Prevention of Key Laboratory of State Ethnic Affairs Commission, Guizhou Minzu University, Guiyang, 550025 China; 2 Department of Applied Ecology, Faculty of Environmental Sciences, Czech University of Life Sciences Prague, Kamýcka 129, Prague-Suchdol, 16500 Czech Republic

**Keywords:** Cd contamination, Selenium, Transcriptome, Proteome, Oryza sativa L

## Abstract

**Background:**

Cadmium (Cd) pollution has declined crop yields and quality. Selenium (Se) is a beneficial mineral element that protects plants from oxidative damage, thereby improving crop tolerance to heavy metals. The molecular mechanism of Se-induced Cd tolerance in rice (Oryza sativa) is not yet understood. This study aimed to elucidate the beneficial mechanism of Se (1 mg/kg) in alleviating Cd toxicity in rice seedlings.

**Results:**

Exogenous selenium addition significantly improved the toxic effect of cadmium stress on rice seedlings, increasing plant height and fresh weight by 20.53% and 34.48%, respectively, and increasing chlorophyll and carotenoid content by 16.68% and 15.26%, respectively. Moreover, the MDA, ·OH, and protein carbonyl levels induced by cadmium stress were reduced by 47.65%, 67.57%, and 56.43%, respectively. Cell wall metabolism, energy cycling, and enzymatic and non-enzymatic antioxidant systems in rice seedlings were significantly enhanced. Transcriptome analysis showed that the expressions of key functional genes psbQ, psbO, psaG, psaD, atpG, and PetH were significantly up-regulated under low-concentration Se treatment, which enhanced the energy metabolism process of photosystem I and photosystem II in rice seedlings. At the same time, the up-regulation of LHCA, LHCB family, and C4H1, PRX, and atp6 functional genes improved the ability of photon capture and heavy metal ion binding in plants. Combined with proteome analysis, the expression of functional proteins OsGSTF1, OsGSTU11, OsG6PDH4, OsDHAB1, CP29, and CabE was significantly up-regulated under Se, which enhanced photosynthesis and anti-oxidative stress mechanism in rice seedlings. At the same time, it regulates the plant hormone signal transduction pathway. It up-regulates the expression response process of IAA, ABA, and JAZ to activate the synergistic effect between each cell rapidly and jointly maintain the homeostasis balance.

**Conclusion:**

Our results revealed the regulation process of Se-mediated critical metabolic pathways, functional genes, and proteins in rice under cadmium stress. They provided insights into the expression rules and dynamic response process of the Se-mediated plant resistance mechanism. This study provided the theoretical basis and technical support for crop safety in cropland ecosystems and cadmium-contaminated areas.

**Supplementary Information:**

The online version contains supplementary material available at 10.1186/s12870-024-05076-7.

## Highlights

Se could improve the growth inhibition of rice under Cd stress.

Se mediated the increase of photosynthetic pigment content.

Se up-regulates functional genes and proteins involved in the photosynthetic system.

Se-mediated decreased the content of MDA, ·OH, and protein carbonyl.

Se-mediated plant hormone signal transduction plays a vital role in Cd detoxification.

**Supplementary Information:**

The online version contains supplementary material available at 10.1186/s12870-024-05076-7.

## Introduction

With the acceleration of industrialization, heavy metal pollution has become a significant environmental concern that limits crop production and threatens the environment and human health [25]. Cadmium (Cd) is a typical and hazardous heavy metal element that can severely affect plant growth and development, even at low concentrations [38]. Due to the rapid development of industrial activities such as mining, leather production, nickel–cadmium batteries production, and electroplating, as well as the increase in anthropogenic activities such as sewage discharge, these are the key sources of Cd pollution in the environment [46]. Plants may uptake and transport Cd from soil to their edible parts via their roots, eventually threatening human health through the food chain, resulting in liver and kidney dysfunction, cancer, and other diseases [58]).

Since plants have abundant non-selective cation channels, Cd^2+^ has good water solubility and mobility and a similar hydration radius to plant essential divalent cation trace elements; Cd^2+^ can easily be absorbed by plants [38]. However, Cd is a harmful heavy metal element that can cause irreversible damage to plants by disrupting the homeostasis of essential elements in plants, causing cellular proteins to replace their positions and interfering with the absorption of essential metal elements such as Cu, Fe, and Zn [41]. Previous studies have shown that significant accumulation of Cd can inhibit plant growth and development, destroy the photosynthetic system, inhibit transpiration, interfere with the metabolic pathways of C, N, and P nutrients, induce the production of large amounts of reactive oxygen species (ROS), aggravate the degree of membrane lipid peroxidation, and lead to abnormal expression of proteins and signal transduction substances [52]. Rice (*Oryza sativa L.*) is one of the most important food crops in China and the world. It is the primary food source for more than 3.5 billion people worldwide and one of China's most important food crops [23, 44]. Rice production is directly related to economic stability and food safety. The use of growth regulators such as salicylic acid and betaine, and various mineral elements, including selenium (Se), zinc (Zn), and silicon (Si), have been studied in recent years [4, 15, 47, 54, 63].

Selenium (Se) is a valuable mineral nutrient element. Compared with Si and Zn, Se has a more prominent role in the regulation of plant physiological responses and is involved in a more comprehensive mechanism [59]. A large number of studies have shown that Se is beneficial to plant growth and development; it can reduce the oxidative damage induced by Cd and regulate the absorption and transport of Cd in plants, thus reducing the accumulation of Cd in crops [11, 33]. At the same time, it can regulate the antioxidant system of plants, increase the activity of glutathione peroxidase (GPX), remove overgenerated reactive oxygen species (ROS) and lipid peroxides (LPO), improve the resistance of plants to stress, enhance the antioxidant properties of plants, and ensure the everyday life activities of plants [62]. In addition, under heavy metal stress, Se can regulate the biosynthesis pathway of porphyrins in plants, accelerate the synthesis of chlorophyll, stimulate plant photosynthesis and the accumulation of starch and sugar to promote plant growth and development [31], restore the ultrastructural disorders caused by heavy metals, and maintain the typical structure and function of plant cells. Examples include chloroplast deformation and loss of thylakoid membrane [38]. At the same time, Se can change the distribution of Cd in the root cell wall and vacuole, inhibit the transfer of Cd, stimulate the production of phytochelatins (PCs), glutathione (GSH), and metallothionein (MT), and promote the functional expression of vacuole transporters. Jointly realize the storage of Cd [41, 52]. In addition, Se can also increase the content of polysaccharides and lignin in the plant body to change the composition of the cell wall, enhance the adsorption capacity of the plant cell wall to Cd, and thus reduce the transfer of Cd to plant cells and aboveground parts [6, 45, 51].

In this study, physiological, biochemical, transcriptional, and proteomic methods were used to reveal the regulation process of Se-mediated critical metabolic pathways, functional genes, and proteins in rice under cadmium stress and to clarify the expression rules and dynamic response process of Se-mediated plant resistance mechanism through plant molecular mechanism studies. The results of this study will provide valuable insights into the molecular intricacies of how Se effectively mitigates Cd-induced toxicity in rice seedlings, providing a deeper understanding of this protective mechanism. In practice, it provides planting optimization means for crop safety production in cropland ecosystems and cadmium-contaminated areas, further understands the effect and function of Se-mediated enhancement of crop production, and finally achieves the purpose of practical application.

## Materials and methods

### Plants and growing conditions

Rice seeds with whole grains and similar size were selected and sterilized with 5% hydrogen peroxide (H_2_O_2_) solution for 15 min and then washed repeatedly with sterile deionized water more than five times. The washed seeds were placed in a petri dish and incubated in an incubator at 25°C for two days in the dark and five days in the light. Rice seedlings with similar growth in the single-leaf stage were selected and transplanted into a pot containing 350 g of sterilized rice soil and then covered with 150 g of sterilized rice soil after uniform distribution. Thirty plants per pot, 3 POTS per treatment. The ambient light intensity of the greenhouse is 176 μmol m^2^ s^−1^, the light/dark period is 14/10 h, the relative humidity is 70% ± 5%, and the temperature is 25°C. Growth conditions were maintained with a 1/4 concentration Hoagland nutrient solution. Design 4 processing: (1)CK: Do not add Cd and Se; (2)Cd: Add 3 mg/kg Cd; (3)L-Se-Cd: Add 3 mg/kg Cd and 1 mg/kg Se; (4)H-Se-Cd: Add 3 mg/kg Cd and 5 mg/kg Se. In the two-leaf stage of rice seedlings, Cd and Se were added to the soil in the form of CdCl_2_ and Na_2_SeO_3_ solutions, respectively, so that the final concentration of Cd in the soil was 3 mg/kg, and the final concentration of Se was 1 mg/kg and 5 mg/kg. The concentration of Cd was selected according to GB 15618–2018 "Soil Environmental Quality—Agricultural Land Soil Pollution Risk Control Standard," and the pH of paddy soil was 6.6. The concentration of Se was screened according to a study by Jiao et al. on the distribution and transport of heavy metals in selenium-rich rice [19]. Each treatment is repeated three times (3 POTS), and the nutrient solution is poured every five days.

### Measurement and sampling of growth parameters

The growth of rice seedlings was recorded every five days and photographed. After 15 days of treatment, destructive plant samples were collected for evaluation assay. Remove most of the soil from the potted plant with a sterile knife, then lift the plant and gently pat it to remove the soil fixed to the root of the plant, and then rinse it slowly with running water to obtain a complete rice plant. The soil was placed on yellow paper, naturally air-dried on an air-drying rack, knocked in a cloth bag, passed through a 100-mesh screen, and put into a sealed bag for the determination of the soil's physical and chemical properties and heavy metal content [60]. Three rice plants with similar growth conditions were selected from each treatment, the fresh weight was determined, and the plants' taproot length and plant height were recorded with a tape measure. The roots and leaves of the plants were isolated, the collected roots were young roots 0.3 cm below the ground, wrapped in tin foil, quickly frozen in liquid nitrogen, and transferred to a refrigerator at -80°C for enzyme-promoted and non-enzyme-promoted antioxidant indices, oxidative stress, transcriptome, and proteome analysis.

### Cd and Se content analysis

The rhizosphere soil, root, and leaf samples were dissolved in a 1:3 v/v mixture of HCL and HNO_3_. When finished, add to a 50 ml volumetric bottle and dilute the mixture with deionized water. In order to test the cadmium content, ICP-MS was used to measure the Cd and Se content of the experimental sample [48].

### The enrichment factor and translocation factor of Cd and Se

Enrichment factor (CCF = total Cd content in rice roots and leaves/total Cd content in soil; SCF = total Se content in rice roots and leaves/total Se content in soil) and transport factors (TCF = Cd content in rice leaves/Cd content in rice roots; TSF = Se content in rice leaf/Se content in rice root [60].

### Analysis of physiological and biochemical indexes of plants

The standard kit of Shanghai Enzyme-Linked Biology (www.mlbio.cn) Technology Co., LTD. Chlorophyll, carotenoid, glutathione (GSH), oxidized glutathione (GSSH), proline (PRO), cysteine (Cys), ascorbate (AsA), soluble sugar, flavonoids, anthocyanins, hydroxyl radical (·OH), malondialdehyde (MDA), protein carbonyl group, hemicelluloses, pectin and Citric were detected acid (CA) content and Total antioxidant capacity (T-AOC). The standard kit of Shanghai Enzyme-Linked Biology (www.mlbio.cn) Technology Co., LTD. The activities of catalase (CAT), peroxidase (POD), superoxide dismutase (SOD), Tyrosine ammoniase (TAL), Succinate dehydrogenase (SDH) were detected.

### Transcriptome analysis

Total RNA was extracted from the tissue using TRIzol® Reagent. Then, RNA quality was determined by 5300 Bioanalyser (Agilent) and quantified using the ND-2000 (NanoDrop Technologies). RNA purification, reverse transcription, library construction, and sequencing were performed at Shanghai Majorbio Bio-pharm Biotechnology Co., Ltd. (Shanghai, China) according to the manufacturer’s instructions (Illumina, San Diego, CA) (www.majorbio.com). Raw reads were cleaned to obtain clean data quickly (https://github.com/OpenGene/fastp). The clean data obtained are then mapped to the reference genome sequence of the rice. Only reads with a perfect match or one mismatch were further analyzed and annotated based on the reference genome. Gene function was annotated based on the following databases: NR (NCBI non-redundant protein sequences), Pfam (Protein family), KOG/eggNOG (Clusters of Orthologous Groups of proteins), Swiss-Prot (a manually annotated and reviewed protein sequence database), KEGG, and GO.

### Proteome profiling

Fresh leaves were ground, suspended, and centrifuged, and the precipitate was extracted as protein. The protein was digested using a filter-aided sample preparation procedure, and the peptide content was estimated. The searches used a peptide mass tolerance of 20 ppm and a production tolerance of 0.02 Da, resulting in a 5% false discovery rate (FDR). The GO annotation proteome classified the proteins derived from the UniProt-GOA database (http://www.ebi.ac.uk/GOA/). The KEGG database was used to annotate the protein pathway. A t-test was used to analyze the expression differences of the proteins between two experimental groups at a threshold value of *P* ≤ 0.05 and FC > 1.5. A two-tailed Fisher’s exact test was employed to test the GO and KEGG pathways and domain enrichment of the differentially expressed proteins against all identified proteins. A correction for multiple hypothesis testing was carried out using standard FDR control methods, with a corrected *P* ≤ 0.05 is considered significant.

### Expression analysis using quantitative RT-PCR

Total RNA (5 µg) isolated from rice shoot exposed to different treatments viz., CK, Cd and CdSeL was reverse transcribed by using SuperScriptII (Fermentas, USA). The synthesized cDNA was diluted in DEPC water in the ratio of 1:5 and subjected to quantitative RT-PCR analysis. Each qRT-PCR reaction was performed in a total reaction volume of 20 μl for each set of selected genes by using Fast SYBR Green PCR Master Mix (Agilent Technologies, USA). The qRT-PCR reactions were performed by using the following cycle conditions: an initial 94°C for 2 min, followed by 30 cycles of 94°C for 30 s, 60°C for 30 s, and 72°C for 30 s, and the final 5 min extension at 72°C. After obtaining the ct-value for each reaction, the relative expression was calculated by 2^-delta Ct method.

### Statistical analysis

One-way ANOVA and IBM SPSS 26.0 (Chicago, USA) were used for statistical analysis. When the assumptions of normal distribution and homogeneity of variance were not satisfied, the non-parametric Kruskal–Wallis test was used to analyze the data. The charts were drawn using Origin 2021 and Adobe Illustrator. Transcriptome and Proteome data were visualized using an online platform (www.majorbio.com).

## Results and analysis

### Rice growth and physiological response

**Fig. 1:**
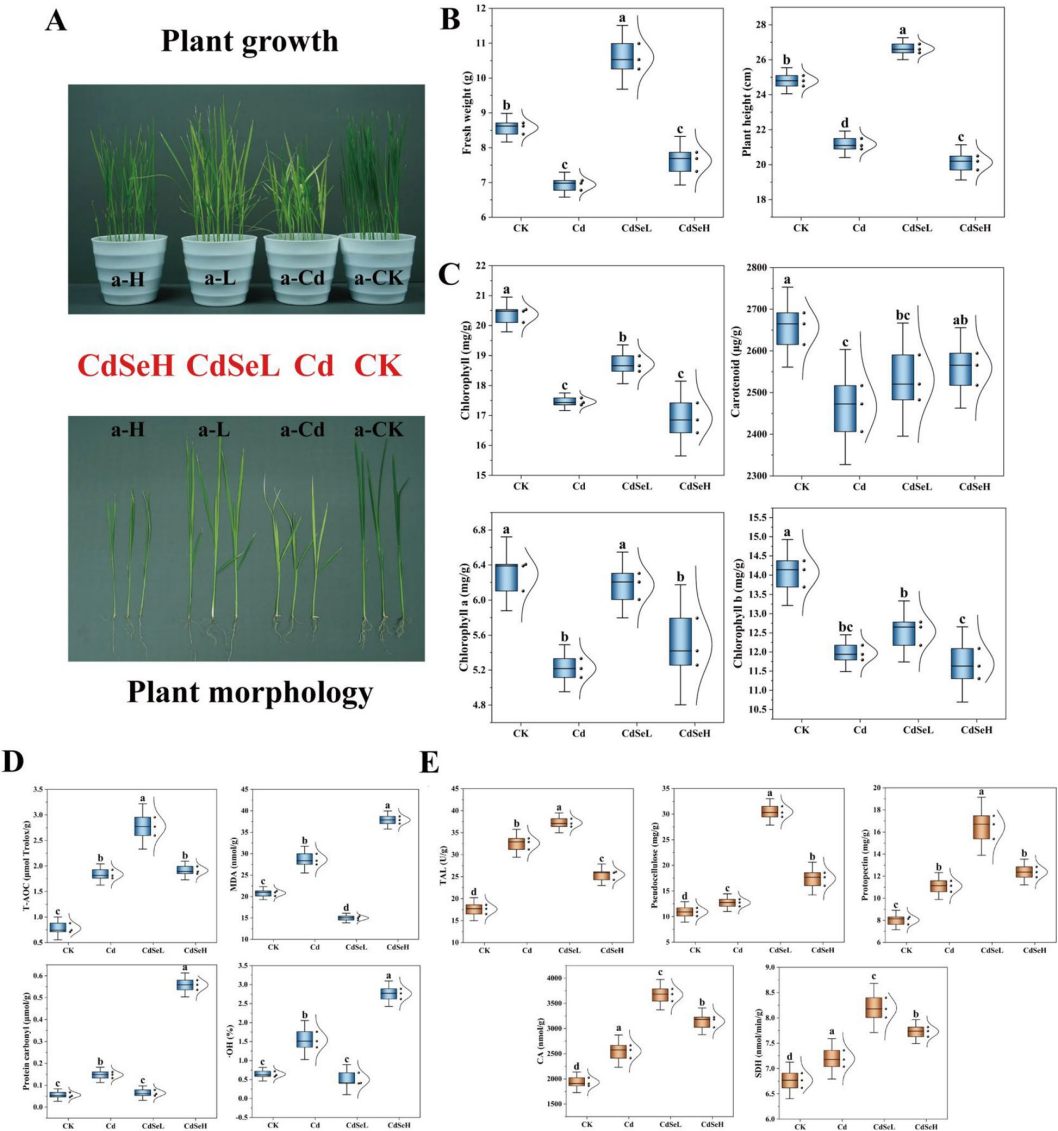
Effects of exogenous Se on rice seedlings' morphological and physiological traits under Cd stress. **A** rice aboveground growth and plant morphology, (**B**) rice fresh weight and plant height, (**C**) photosynthetic pigment content, (**D**) plant oxidative stress level, (**E**) plant somatic cell wall and energy metabolism. The results were mean ± standard deviation values (*n* = 3). One-way ANOVA was used to compare the significant differences in treatments (CK: control, Cd: 3 mg/kg Cd, CdSeL: 3 mg/kg Cd + Se 1 mg/kg, CdSeH: 3 mg/kg Cd + Se 5 mg/kg). Different lowercase above the bars indicated significant differences at the level of *P* < 0.05

Under the stress of Cd, the growth of rice seedlings was notably hindered, characterized by reduced aboveground height, yellowing leaves, and a significant decrease in fresh weight (Fig. 1A, B). Conversely, the introduction of low-concentration Se (CdSeL treatment) significantly improved the adverse growth induced by Cd stress, leading to robust growth, increased height, and greener leaves. Plant height, fresh weight and photosynthetic pigment content of rice seedlings were significantly decreased under Cd stress (Fig. 1C). At the same time, the oxidative damage of rice seedlings was aggravated (Fig. 1D). CdSeL treatment significantly reduced the toxicity induced by Cd. This was evident in the 20.53% increase in plant height and a substantial 34.48% rise in fresh weight (Fig. 1B). Furthermore, chlorophyll and carotenoid content increased by 16.68% and 15.26%, respectively (Fig. 1C), and T-AOC continued to increase by 33.91%. However, MDA, ·OH, and protein carbonyl contents decreased significantly by 47.65%, 67.57%, and 56.43%, respectively (*P* < 0.05) (Fig. 1D). In addition, TAL activity, hemicellulose, and pectin contents continued to increase by 12.44%, 58.19%, and 32.75%, while SDH activity and CA content increased by 12.24% and 30.49%, respectively (Fig. 1E). These results collectively indicate that CdSeL treatment effectively alleviated the toxicity caused by Cd stress in rice seedlings, leading to improved growth and reduced oxidative damage.

### Response of rice seedlings to antioxidant stress

**Fig. 2:**
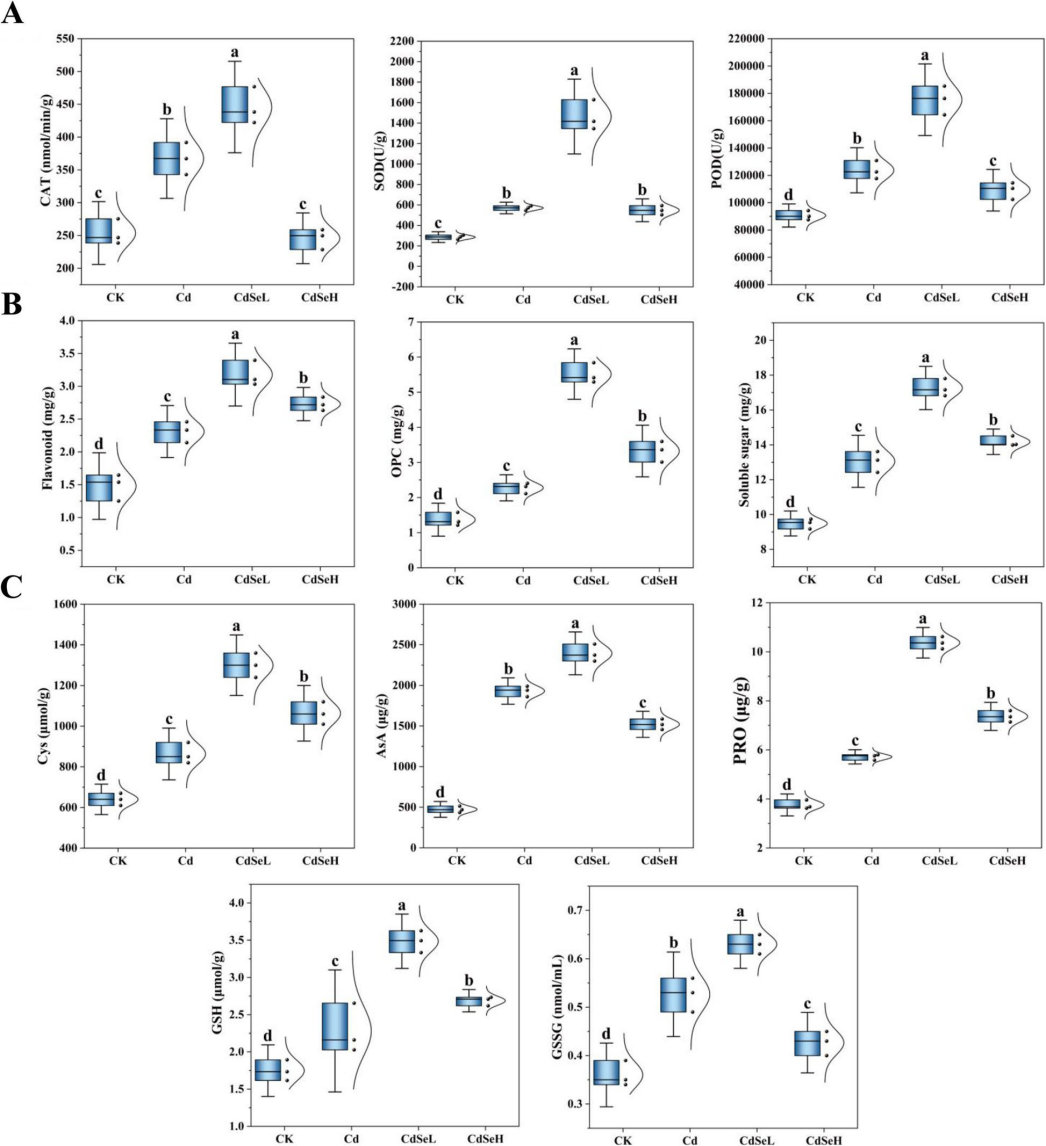
Exogenous Se-induced catalase (CAT), superoxide dismutase (SOD) and peroxidase (POD) activities, flavonoids, anthocyanins (OPC), soluble sugar, cysteine (Cys), ascorbic acid (AsA), proline (PRO), reduced glutathione (GSH) and oxidized glutathione in rice seedlings leaves under Cd stress Effect of glycine (GSH) content. One-way ANOVA was used to compare the significant differences in treatments (CK: control, Cd: 3 mg/kg Cd, CdSeL: 3 mg/kg Cd + Se 1 mg/kg, CdSeH: 3 mg/kg Cd + Se 5 mg/kg). Different lowercase above the bars indicated significant differences at the level of *P* < 0.05

Cd stress significantly reduced the activities of antioxidant enzymes in rice seedlings, among which catalase (CAT), superoxide dismutase (SOD), and peroxidase (POD) activities were reduced by 31.12%, 49.99%, and 26.77%, respectively (Fig. 2A). Following the CdSeL treatment, the antioxidant stress response in rice seedlings was notably strengthened, leading to a reduction in Cd-induced toxicity. Specifically, the activities of CAT, SOD, and POD were significantly increased by 43.19%, 80.50%, and 48.32%, respectively (Fig. 2A). The contents of flavonoids, OPC, and soluble sugar continued to increase by 27.31%, 58.72%, and 24.36% (Fig. 2B). In contrast, the contents of Cys, AsA, PRO, GSH, and GSSG significantly increased by 33.59%, 19.36, 43.88%, 34.55%, and 16.40%, respectively (Fig. 2C). These findings underscore the positive impact of CdSeL treatment on reinforcing the antioxidant defense mechanisms in rice seedlings, ultimately mitigating the toxic effects of Cd stress.

### Accumulation and transport of Se and Cd in rice seedlings

The enrichment factors (CCF and SCF) and transport factors (TCF and TSF) of Cd and Se in plants were calculated by combining rice's underground and aboveground parts. Compared with the CK group, Cd content in soil was significantly increased by 82.14%, Cd content in roots and leaves of rice seedlings was significantly increased by 88.02% and 93.36%, respectively, with CCF reaching 35.2 and TCF 0.102, and the content in roots was much higher than that in leaves. It is worth noting that Cd accumulation in the roots was considerably higher than in the leaves. Following the exogenous addition of Se, rice seedlings' Cd absorption and enrichment capacity were significantly increased. After CdSeL treatment, the Cd content in roots and leaves was significantly increased by 68.15% and 59.91%, respectively, compared to the Cd group (Table 1). This suggests that Se supplementation enhanced the ability of rice seedlings to absorb and accumulate Cd. Conversely, when subjected to high-concentration Se treatment (CdSeH), the Cd absorption and enrichment capacity of rice seedlings began to decline, and the Cd content in roots and leaves decreased significantly by 24.37% and 69.91%, respectively, in comparison to the CdSeL group. However, it is important to note that the Se concentration in the soil increased significantly by 42.99% (Table 1). These results indicate that high Se concentrations may inhibit the uptake and accumulation of Cd in rice seedlings.

**Table 1:** Accumulation and transport of heavy metals

(mg/kg)	Cd (Soil)	Se (Soil)	leaf (Cd)	Root (Cd)	leaf (Se)	Root (Se)	CCF	SCF	TCF	TSF
CK	0.25 ± 0.035c	0.25 ± 0.010c	0.29 ± 0.014c	5.15 ± 0.240d	0.0017 ± 0.00007c	0.0075 ± 0.00014c	21.46 ± 2.09d	0.032 ± 0.00177c	0.056 ± 0.005bc	0.22 ± 0.005b
Cd	1.40 ± 0.106ab	0.21 ± 0.004c	4.37 ± 0.430b	43.0 ± 2.830c	0.0011 ± 0.00007c	0.0049 ± 0.00007c	35.21 ± 0.76c	0.023 ± 0.00014c	0.102 ± 0.017a	0.21 ± 0.011b
CdSeL	1.23 ± 0.077b	1.83 ± 0.060b	10.9 ± 0.850a	135.0 ± 2.82a	0.1450 ± 0.00707a	0.4400 ± 0.01414a	121.3 ± 5.55a	0.386 ± 0.00778a	0.081 ± 0.008ab	0.33 ± 0.027a
CdSeH	1.48 ± 0.014a	3.21 ± 0.150a	3.28 ± 0.226b	102.1 ± 6.93b	0.0885 ± 0.00071b	0.3850 ± 0.00707b	72.25 ± 4.25b	0.209 ± 0.00849b	0.032 ± 0.001c	0.23 ± 0.002b

Accumulation and transport of heavy metal ions in rice roots and leaves. One-way ANOVA was used to compare the significant differences of treatments (CK: control, Cd: 3 mg/kg Cd, CdSeL: 3 mg/kg Cd + Se 1 mg/kg, CdSeH: 3 mg/kg Cd + Se 5 mg/kg). Different lowercase above the bars indicated significant differences at the level of *P* < 0.05

### Enrichment of differentially expressed functional genes in rice seedlings

**Fig. 3:**
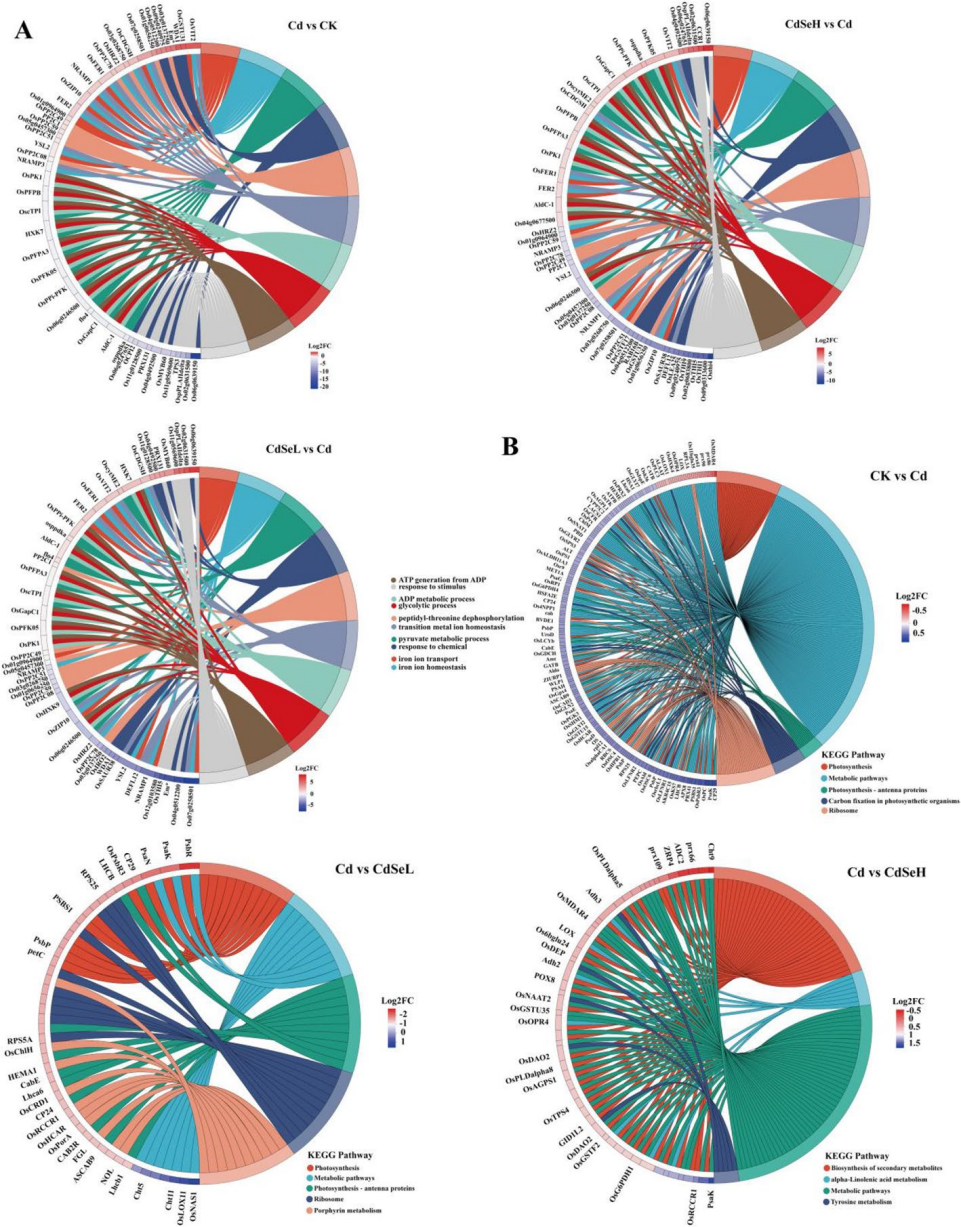
Enrichment diagram of GO and KEGG differentially expressed functional genes. **A** DEGs in GO enrichment analysis, and (**B**) DEGs in KEGG enrichment analysis. (CK: control, Cd: 3 mg/kg Cd, CdSeL: 3 mg/kg Cd + Se 1 mg/kg, CdSeH: 3 mg/kg Cd + Se 5 mg/kg)

In the transcriptomic analysis, a total of 77.17 Gb of Clean Data was obtained from the 12 samples. The Clean Data for all samples exceeded 6.04 Gb, and the percentage of Q30 bases was above 94.81% (Table S1). There were 80 common differentially expressed functional genes (DEGs) identified in the three comparison groups, and there was a distinct pattern of differences among these groups. Specifically, in the CK vs Cd comparison group, 533 DEGs were identified. After low-concentration Se treatment, the number of DEGs was significantly increased compared with that in the Cd group, and 1688 DEGs were upregulated (Figure S1, Table S2). In the GO enrichment analysis, in the Cd vs CdSeL comparison group, The top 5 DEGs were OsMYB60, PRX131, OsCDGSH, HXK7, and OscytME2, which were mainly involved in glycolysis, heavy metal ion transport, and stress response processes (Fig. 3A). In the KEGG enrichment analysis, in the Cd vs CdSeL comparison group, the top 5 DEGs were PsbR, PsaK, PsaN, CP29, and LHCB. They were mainly involved in photosynthesis and functional expression of photosynthetic proteins (Fig. 3B).

### GO and KEGG pathway enrichment analysis of DEGs in rice seedlings

**Fig. 4:**
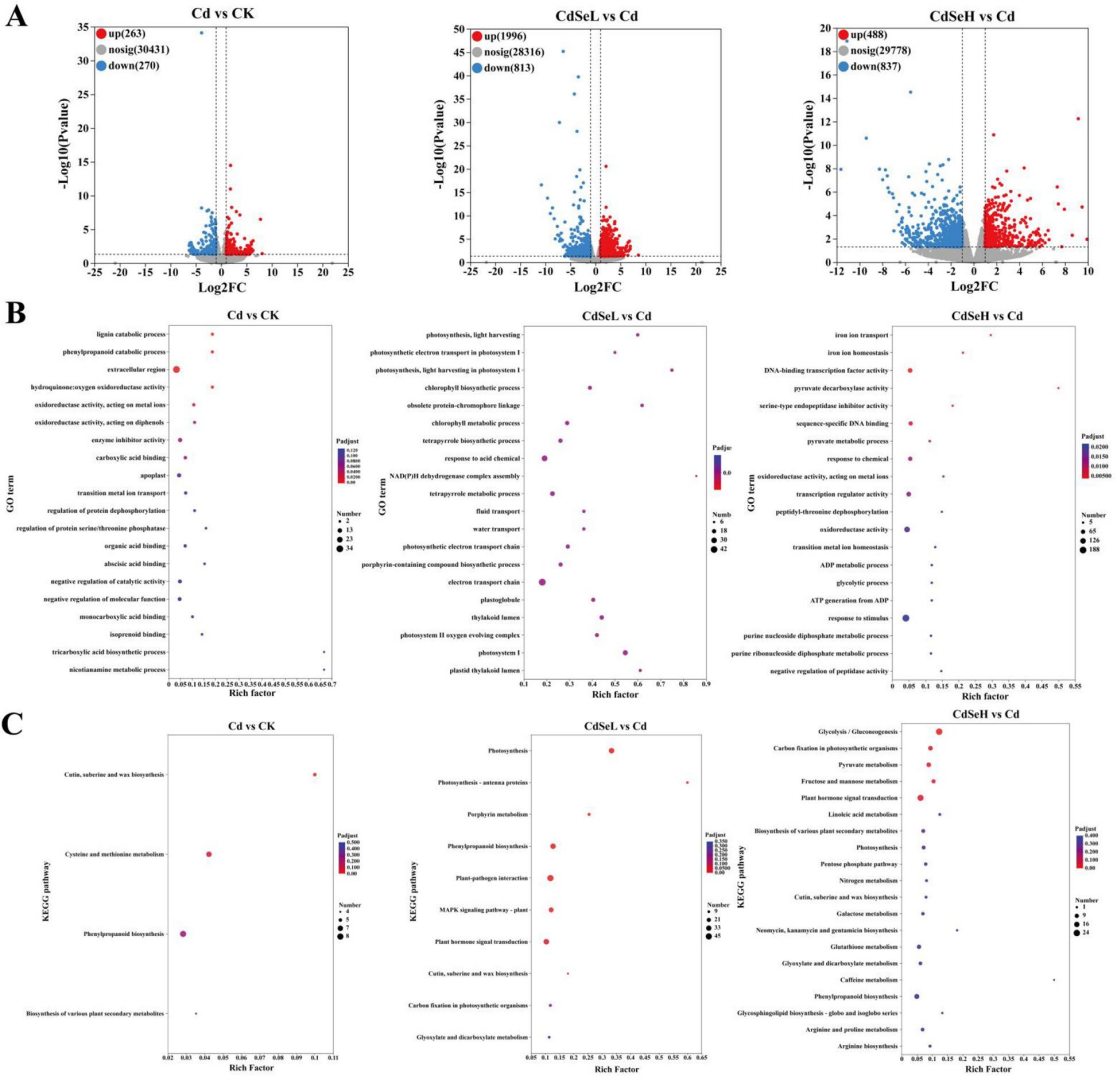
Distribution and enrichment analysis of DEGs. **A** Volcano plots of DEGs up-regulation and down-regulation, (**B**) Enrichment analysis of GO functional pathways, (**C**) The top 20 pathways of the significance of the up-regulated and down-regulated DEGs on KEGG. The X-axis represented the rich factor, and the Y-axis represented the pathway’s name. The bubble size represents the number of DEGs involved. The bubble color indicates the enrichment degree of the pathway. (CK: control, Cd: 3 mg/kg Cd, CdSeL: 3 mg/kg Cd + Se 1 mg/kg, CdSeH: 3 mg/kg Cd + Se 5 mg/kg)

Under Cd stress, a total of 533 DEGs (263 up-regulated, 270 down-regulated) were identified in the CK vs Cd comparison group, and 2809 DEGs (1996 up-regulated, 813 down-regulated) were identified in the Cd vs CdSeL comparison group. In the Cd vs CdSeH comparison group, the expression of DEGs showed a highly significant downward trend. A total of 1325 DEGs (488 up-regulated and 837 down-regulated) were identified (Fig. 4A) and visualized by clustering heat maps (Figure S4). GO enrichment analysis showed that in comparing the Cd vs CdSeL comparison group, DEGs are mainly involved in the electron transport chain, photosynthesis, photosynthetic electron transport in photosystem I, and light harvesting photosystem I, tetrapyrrole biosynthetic process, NAD(P)H dehydrogenase complex assembly, porphyrin-containing compound biosynthetic process, and photosystem II oxygen-evolving complex process. KEGG enrichment analysis showed that DEGs are mainly involved in Cutin, suberine, wax biosynthesis, and Cysteine and methionine metabolism in the CK vs Cd group. In the Cd vs CdSeL comparison group, DEGs are mainly involved in Photosynthesis, Photosynthesis-antenna proteins, Porphyrin metabolism, Phenylpropanoid biosynthesis, and MAPK signaling pathway-plant, Plant hormone signal transduction, and Cutin, suberine, and wax biosynthesis processes.

### Key pathways of DEGs involvement in rice seedlings

**Fig. 5:**
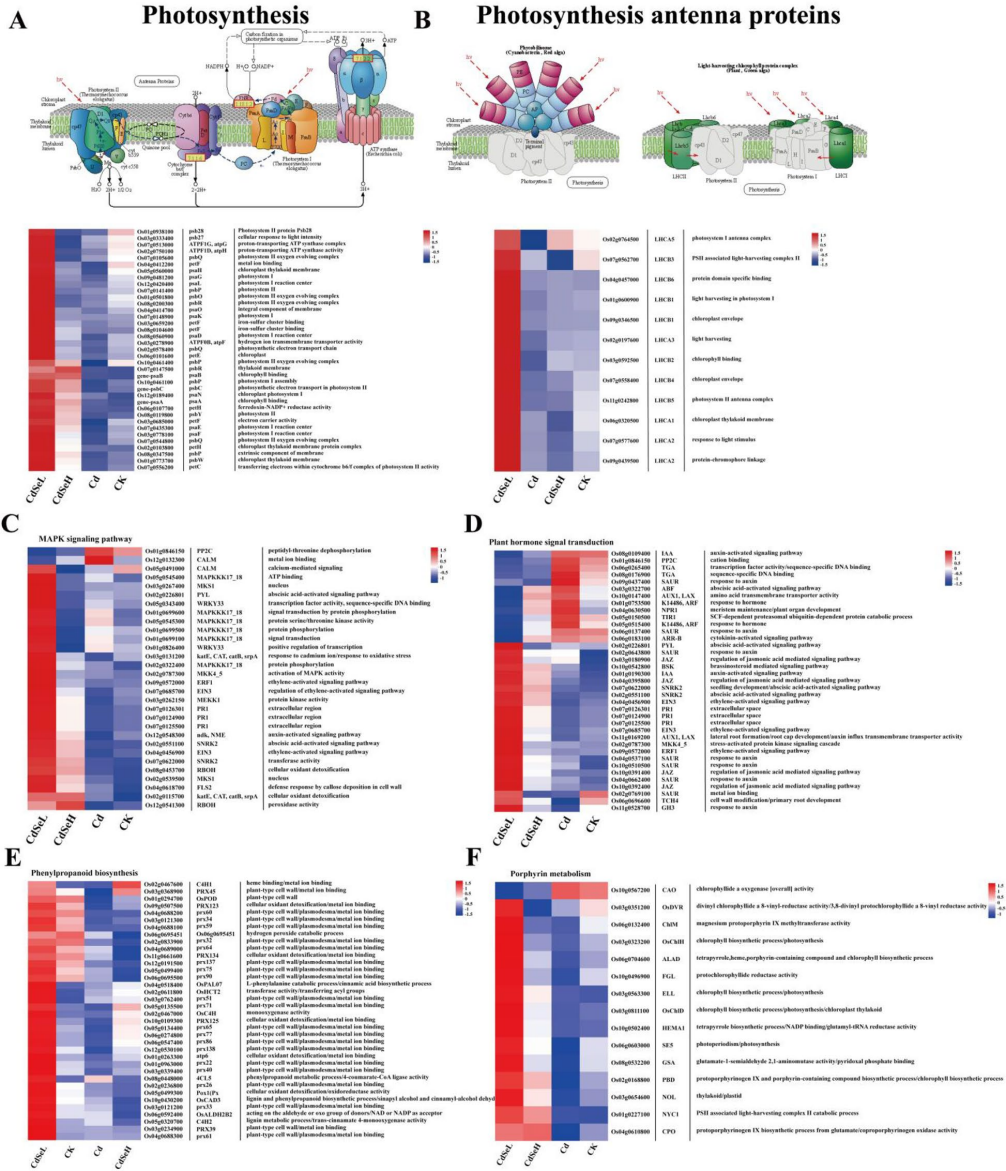
Expression level of DEGs in rice leaves under Cd stress. **A** Photosynthesis, (**B**) Photosynthesis—antenna proteins, (**C**) MAPK signaling pathway—plant, (**D**) Plant hormone signal transduction, (**E**) Phenylpropanoid biosynthesis, (**F**) Porphyrin metabolism. (CK: control, Cd: 3 mg/kg Cd, CdSeL: 3 mg/kg Cd + Se 1 mg/kg, CdSeH: 3 mg/kg Cd + Se 5 mg/kg)

This study examined the impact of Se on several key gene pathways, including in photosynthesis, plant hormone signal transduction, phenylpropanoid biosynthesis, and porphyrin metabolism were affected by Se. After the exogenous addition of low-concentration Se(CdSeL), the functional genes of photosynthesis were significantly upregulated compared with those in the Cd group (Fig. 5A). At the same time, the expression of genes encoding photosynthetic functional proteins (LHCA and LHCB families) was also significantly upregulated by CdSeL (Fig. 5B). In the signal transduction pathway, CdSeL-mediated encoding of plant hormones (PYL, EIN3, EAF1, SNRK2, NME, IAA, ABF, and JAZ), oxidative stress response to Cd ions (katE, CAT, catB, and srpA), energy cycling (MAPKKK17_18), cell wall ion deposition (FLS2), and phytogenesis The functional genes of long development (NPR1, AUX1, LAX, and TCH4) were significantly upregulated (Fig. 5C, D). In phenylpropanoid biosynthesis, CDSEL-mediated functional genes encoding heavy metal ion binding (C4H1, PRX family, and atp6), cell wall synthesis (OsPAL07, 4CL5, OsCAD3, and C4H2), and oxidative detoxification (Pox1) were significantly upregulated (Fig. 5E). Finally, in porphyrin metabolism, CDSEL-mediated effects resulted in the upregulation of genes associated with chlorophyll biosynthesis (OsChlH, ALAD, and ELL) and the synthesis of pigment precursor substances for photosynthetic pigments (ChlM, HEMA1, PBD, and CPO) (Fig. 5F). These findings suggest that Se plays a significant role in enhancing various pathways related to photosynthesis and stress response in rice under Cd stress.

### Weighted gene co-expression network analysis (WGCNA)

**Fig. 6:**
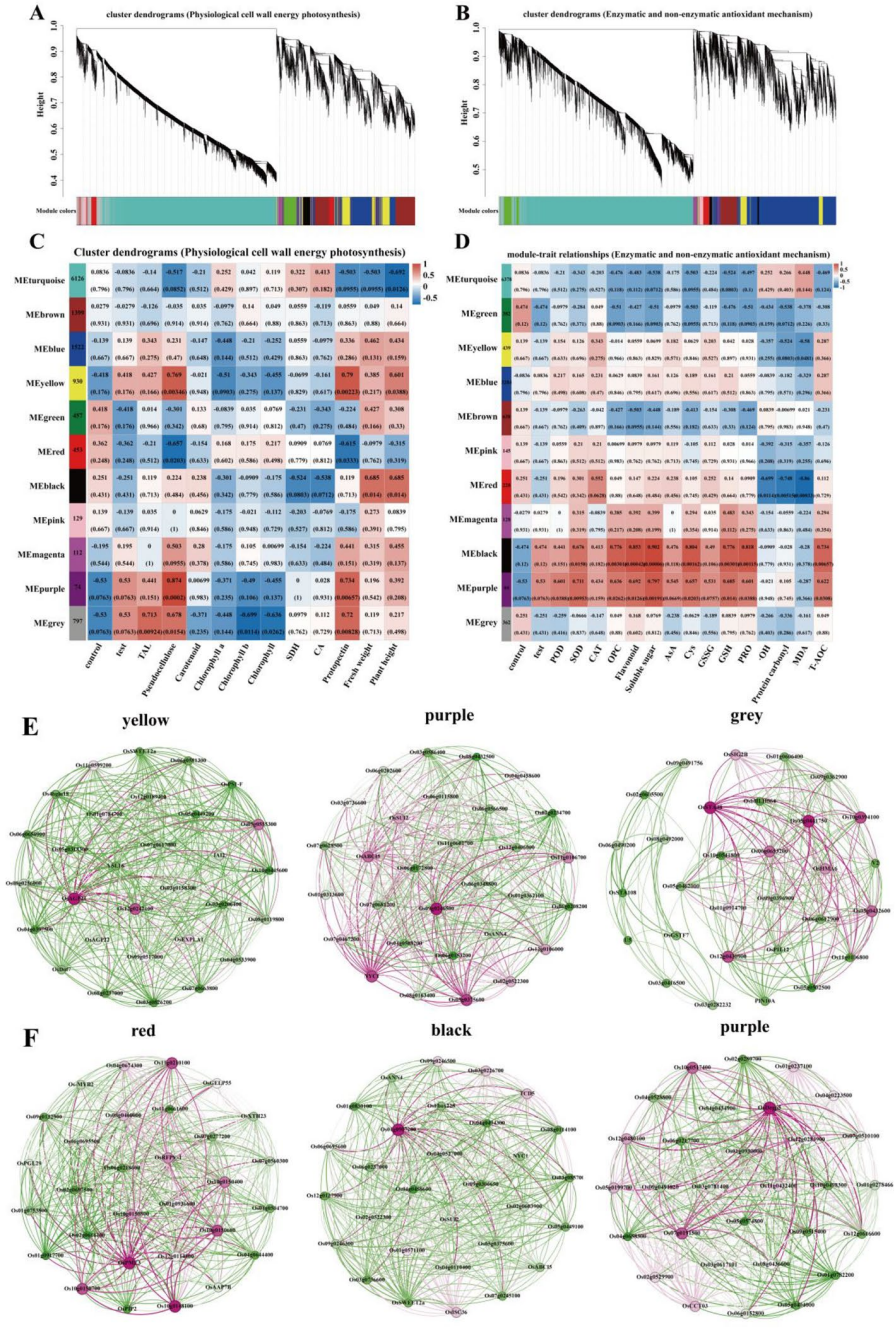
Results of the weighted gene co-expression network analysis (WGCNA) and Symbiotic network results for the mRNA expression profiles of differentially expressed genes (DEGs). The cluster dendrograms DEGs and assigned colors for leaf (**A** and **B**), The relationships between sample indexes and co-expression module in leaf (**C** and **D**). **E** Color module functional gene network analysis with a highly significant positive correlation with physiological traits index, and (**F**) color module functional gene network analysis with extremely significant positive correlation with oxidative stress index. (CK: control, Cd: 3 mg/kg Cd, CdSeL: 3 mg/kg Cd + Se 1 mg/kg, CdSeH: 3 mg/kg Cd + Se 5 mg/kg)

Based on RNA-seq data, co-expression network analysis was conducted to study the critical genes related to biological indicators. As shown in Fig. 6A and B, the screened functional genes were divided into 11 modules, and correlation analysis was conducted with physiological and oxidation indicators, respectively. Among them, yellow, purple, and gray modules were significantly positively correlated with physiological indices (TAL, hemicelluloses, and protection), and red, purple, and black modules were significantly positively correlated with antioxidant indices, among which the gene clusters of each module were highly correlated (Fig. 6C, D). These key modules were then analyzed, and the GO enrichment analysis showed that, Among the physiological indicators, the yellow module enriched participation in chloroplast thylakoid, plastid thylakoid membrane, thylakoid membrane, photosynthetic membrane, and plastid chloroplast pathways. Purple modules enrich participation in Photosynthesis, photosynthetic electron, chlorophyll-binding, oxidoreductase activity, and ferroxidase. The gray module enriches the pathways involved in transition metal ions (Figure S5). Through co-expression network analysis, The essential genes OsAGP21, YSL16, OsABC15, NYC1, OsSUI2, OsSTA38, OsPME3, OsRFPV-1, Os01g0907200, and OsDegp5 with the highest connectivity among the six modules were found (Fig. 6E, F).

### Proteomic analysis of rice seedlings

**Fig. 7:**
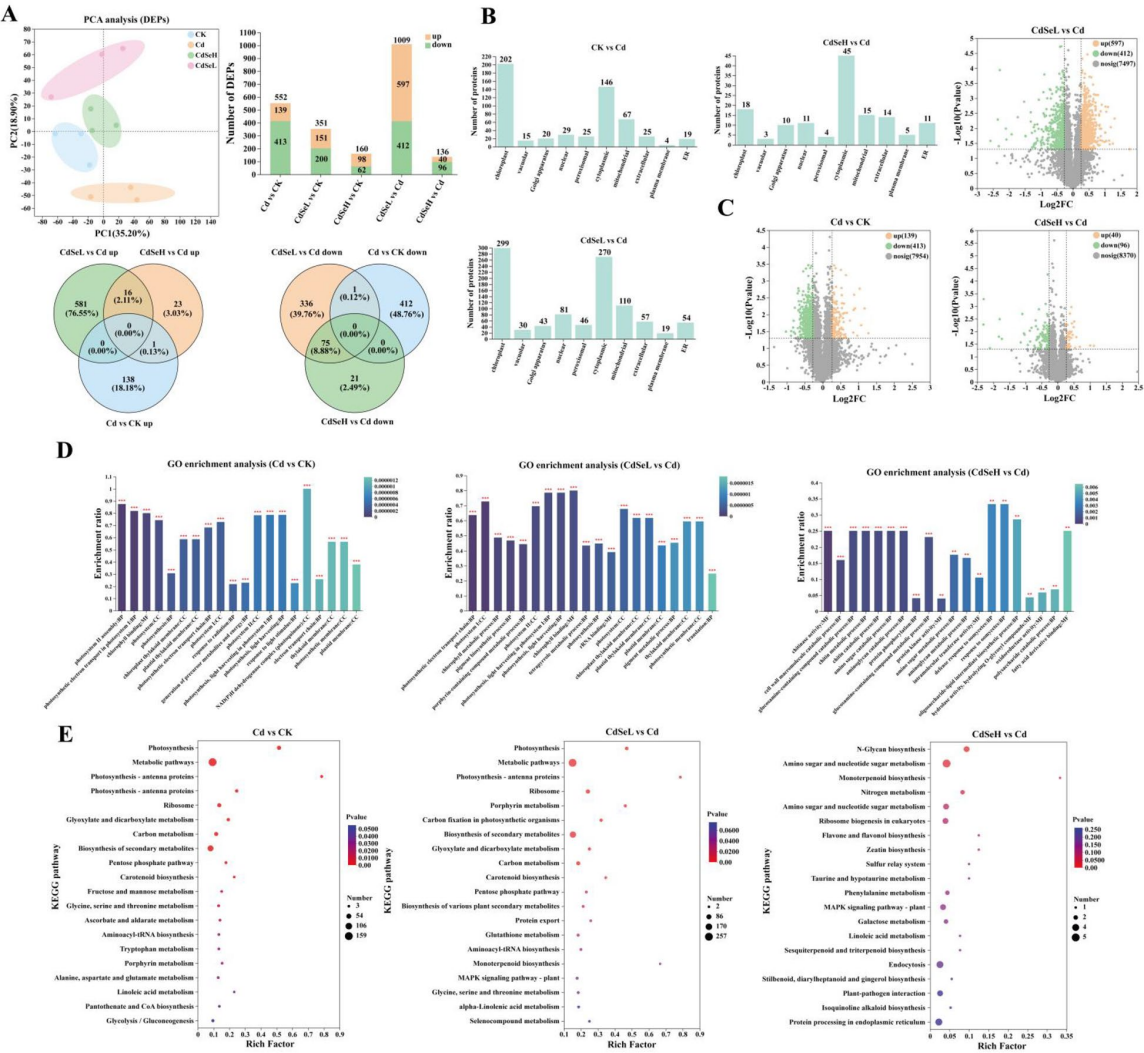
Proteomic analysis. **A** DEPs PCA analysis, DEPs stacking diagram, DEPs Veen diagram, (**B**) Subcellular localization analysis of DEPs, (**C**) Volcano plots of DEPs up-regulation and down-regulation, (**D**) DEPs GO enrichment analysis, (**E**) DEPs KEGG enrichment analysis. (CK: control, Cd: 3 mg/kg Cd, CdSeL: 3 mg/kg Cd + Se 1 mg/kg, CdSeH: 3 mg/kg Cd + Se 5 mg/kg)

We further investigated the changes in protein expression in rice to understand the mechanism of SE-mediated relief in rice seedlings under Cd stress. A total of 8506 proteins were identified, among which 2396 were differentially expressed proteins (DEPs), and there were apparent protein expression partitions among the treatment groups (Fig. 7A, C). In addition, we identified the subcells involved in DEPs, mainly in chloroplast and cytoplasmic in the CK vs Cd group. CdSeL vs Cd was mainly concentrated in mitochondrial, chloroplast, nuclear, and cytoplasmic (Fig. 7B). GO analysis shows that in the CdSeL vs Cd comparison group, DEPs are mainly involved in chloroplast thylakoid membrane, plastid thylakoid membrane, pigment metabolic process, and photosynthetic metabolic process of membrane and thylakoid membrane (Fig. 7D, Figure S7). KEGG analysis showed that in the CdSeL vs Cd comparison group, DEPs are mainly involved in porphyrin metabolism, carbon fixation in photosynthetic organisms, biosynthesis of secondary organisms metabolites, glyoxylate and dicarboxylate metabolism, carbon metabolism, carotenoid biosynthesis, pentose phosphate pathway, Photosynthesis and Ribosome processes. Specifically, they were prominently involved in N-Glycan biosynthesis, amino sugar and nucleotide sugar metabolism, monoterpenoid biosynthesis, and nitrogen metabolism. Moreover, DEPs were associated with amino sugar and nucleotide sugar metabolism, as well as ribosome biogenesis in eukaryotes, as indicated in Fig. 7E and Figure S7).

### Analysis of DEPs interaction network in rice seedlings

**Fig. 8:**
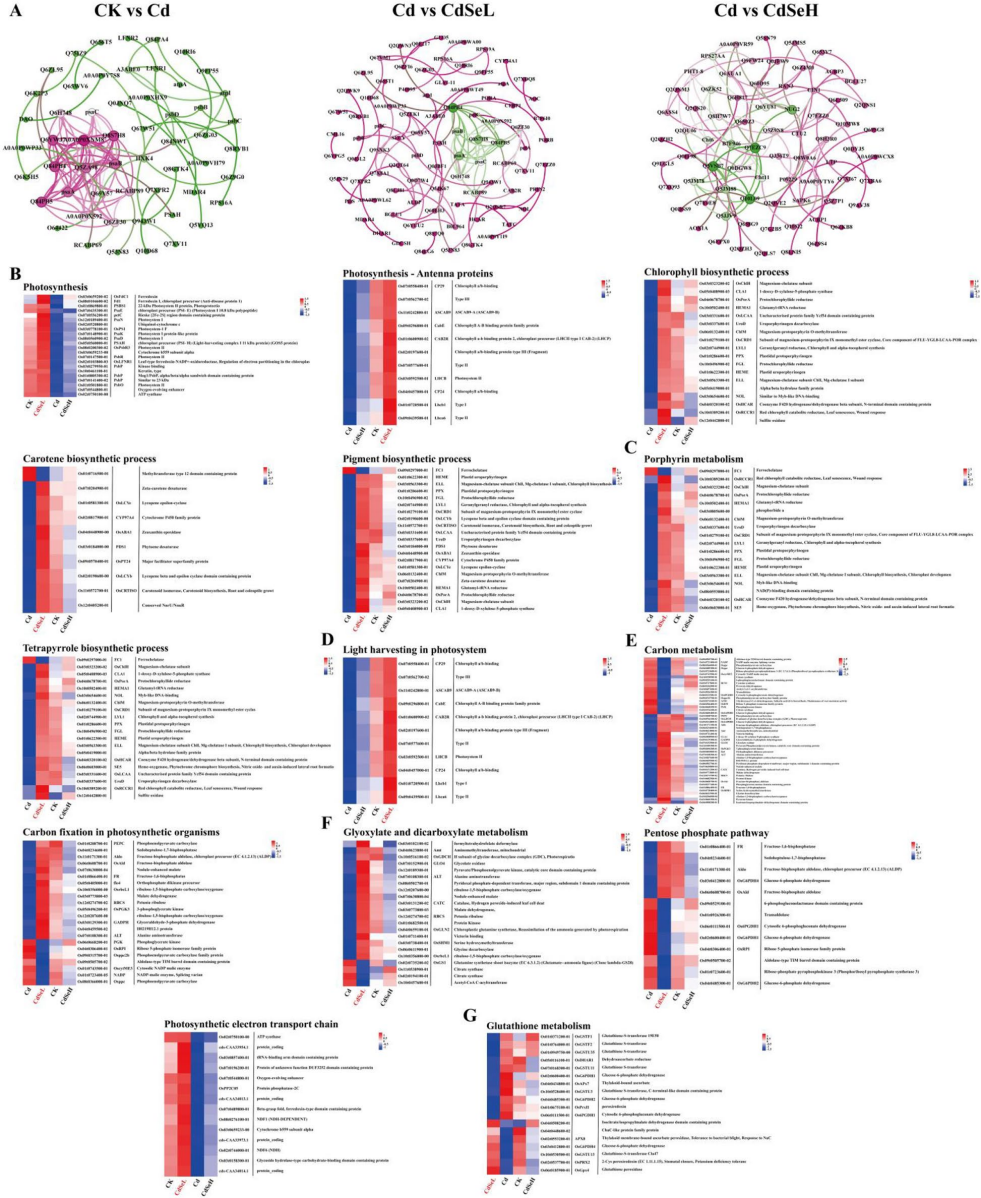
Expression levels of DEPs in rice leaves under Cd stress. **A** Analysis of the symbiotic network of differentially expressed proteins in each comparison group, and (**B**) heat maps of DEPs correlation in photosynthetic pigment biosynthesis, (**C**) Porphyrin and tetrapyrrole biosynthetic process, (**E**) Carbon fixation and Carbon metabolism, (**F**) Glyoxylate and dicarboxylate metabolism and Pentose phosphate pathway, (**G**) Glutathione metabolism. (CK: control, Cd: 3 mg/kg Cd, CdSeL: 3 mg/kg Cd + Se 1 mg/kg, CdSeH: 3 mg/kg Cd + Se 5 mg/kg)

In this study, critical hub genes involved in regulation in each comparison group were identified through network analysis. Here's a summary of the key functional proteins and their roles in different groups: (1) CK vs. Cd comparison group: Critical functional proteins: Q84PB4, Q5ZA98, Q8S7H8, Q84PB5, psaA, psaB, Q6YWJ7, and A0A0P0XNM8. Notable Effects: Under Cd stress, the functional proteins associated with photosystem I (PsaN, PsaE, OsPS1, PsaK, PsaD, and PSAH), photosystem II (PSBS1, OsPsbR3, PsbR, and PsbO) and oxidoreductase (OsFdC1, Fd1, OSFDC1, OsLFNR1) were significantly down-regulated. (2) CdSeL vs Cd Comparison Group: Critical Functional Proteins: Q84PB4, Q8S7H8, Q84PB5, psaA, psaB, psaC, Q6H748. Notable Effects: After CdSeL treatment, almost all functional proteins related to photosynthesis were significantly upregulated, except for OsFdC1 and Fd1. Functional proteins encoding chlorophyll a/b binding (CP29, CabE, CAB2R, CP24) were also significantly upregulated. Genes involved in chlorophyll biosynthesis, carotenoid biosynthesis, and photosynthetic pigment synthesis were upregulated, as were genes associated with porphyrin metabolism and tetrapyrrole biosynthesis (Fig. 8). Overall, the study reveals the impact of Se treatment on the regulation of key functional proteins involved in photosynthesis, plant hormone signal transduction, phenylpropanoid biosynthesis, porphyrin metabolism, photon capture, carbon metabolism, energy metabolism, and oxidative stress. CdSeL treatment led to the upregulation of many of these functional proteins, suggesting that Se plays a crucial role in enhancing these pathways and mitigating the effects of Cd stress in rice.

### qRT-PCR analysis results

**Fig. 9:**
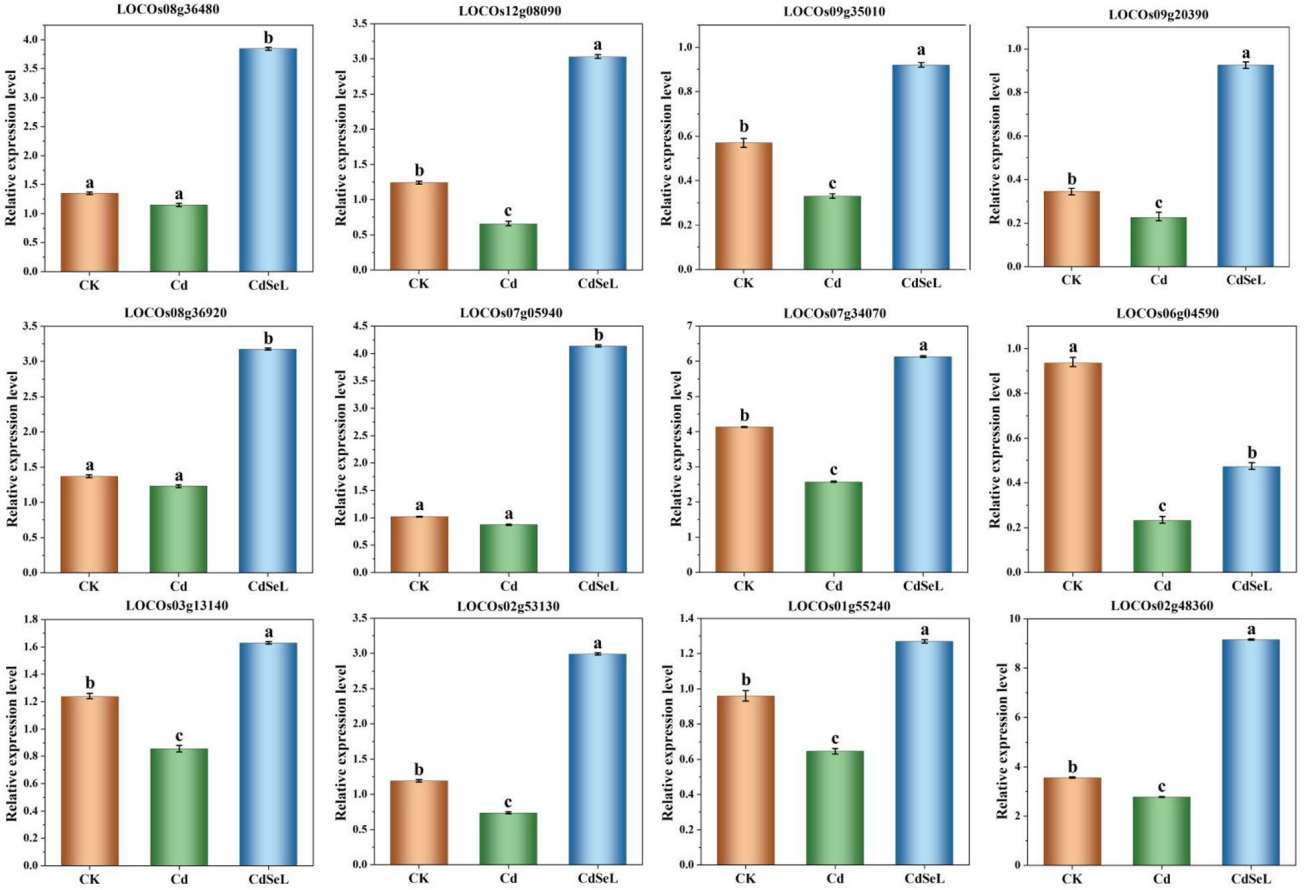
Results of the quantitative PCR (qRT-PCR) results for the mRNA expression profiles of differentially expressed genes (DEGs). (CK: control, Cd: 3 mg/kg Cd, CdSeL: 3 mg/kg Cd + Se 1 mg/kg)

To validate the reliability of RNA-Seq data, we selected 12 DEGs and verified them by qRT-PCR. The similar expression trends of selected DEGs, except in one genes(LOC_Os06g04590), were consistent with the Illumina sequencing, indicating the dependability of the RNA-Seq data (Fig. 9).

## Discussion

### Exogenous Se supplementation improved the growth of rice seedlings and their tolerance to cadmium stress

The accelerated pace of industrialization has resulted in the discharge of various heavy metals into the environment, posing a significant threat to environmental health. Excessive levels of Cd can induce oxidative damage in plants, leading to the generation of reactive oxygen species (ROS) such as superoxide anion (·O_2_) and hydroxyl radicals (·HO). This, in turn, exacerbates the peroxidation of intracellular proteins and membrane lipids, damages the ultrastructure of sub-organelles, and interferes with the absorption of mineral nutrients by roots to inhibit plant growth and development [16, 28, 34, 42, 46, 50, 57, 61]. In this study, exposure to Cd stress at a concentration of 3 mg/kg had several adverse effects on rice seedlings. It inhibited biomass assimilation, interfered with plant development, disrupted the photosynthetic system, and intensified oxidative damage, all of which negatively impacted rice seedling growth (Fig. 1A-D). Previous research has indicated that rice seedlings possess a significant number of non-selective cation channels that facilitate the uptake of Cd^2+^. Cd^2+^ is highly soluble in water and shares a similar hydration radius with essential bivalent cation trace elements found in plants. Therefore, Cd^2+^ can easily be absorbed by rice seedlings [38]. However, excessive accumulation of Cd^2+^ can impede rice seedlings' transpiration, disrupt metabolic pathways related to C, N, and P nutrients, and induce the production of a large number of ROS, leading to abnormal expression of proteins and signal transducers, destroy homeostasis of essential elements in plants, make cell proteins replace their positions, and interfere with the absorption of Cu, Fe, Zn and other essential metal elements. Thus, the growth and development of rice seedlings were negatively affected [49]. As a non-essential but beneficial element in plants, Se is considered to have a potential role in protecting plants from environmental abiotic stresses [19]. In this study, CdSeL treatment increased the root system to absorb the Cd. However, it limited the Cd to the ground part of the transfer, improved the activity of TAL, and increased the content of photosynthetic pigment, hemicellulose, and pectin, thus enhancing the rice seedlings to the Cd stress tolerance, promoting the assimilation and accumulation of rice seedling biomass (Fig. 1, Table 1). In addition, SE-mediated AsA-GSH cycle, GSH-GSSG cycle, antioxidant enzyme activity, and non-enzymatic antioxidant content also play a crucial role in clearing excess ROS induced by Cd, protecting organelles and cell structures in rice seedlings from oxidative damage (Fig. 2). Rao et al. revealed the mechanism of action of selenates on Cardamine violifolia through multi-omics studies, and the results showed that a low concentration of Se could regulate the biosynthesis pathway of porphyrins in plants and accelerate the synthesis of chlorophyll, stimulate plant photosynthesis and the accumulation of starch and sugar to promote plant growth and development [31, 32]. In addition, low concentrations of Se were shown to modulate the antioxidant system in plants, increasing the activity of glutathione peroxidase (GPX) to eliminate excessive ROS production effectively. This enhances plant stress and resistance, fortifies their antioxidant defenses, and ensures their normal metabolic activities [9, 10].

### The addition of exogenous Se improved the photosynthesis of rice seedlings under Cd stress

Indeed, it has been widely documented that Se can improve plant photosynthesis and promote biomass assimilation, especially under abiotic stress conditions [43]. In this study, it is evident that CdSeL treatment significantly improved the photosynthesis of rice seedlings. Se played a key role in regulating various components of the photosynthetic apparatus in rice seedlings, including photosystem II (PSII), photosystem I (PSI), photosynthetic electron transport, and photon capture proteins (LHCA and LHCB families) (Figs. 3, 4 and 5). Photosystem II (PSII) is a critical component of the photosynthetic machinery in plants located in the stroma membrane of chloroplast. It consists of multiple protein subunits and pigment molecules that capture light energy and convert it into chemical energy while releasing electrons from water molecules and producing oxygen during light energy capture [40]. In addition, previous studies have shown that PsbR protein can optimize the electron transfer and water oxidation processes in plants, enhance the role of oxygen evolution, and repair the PSII system [25, 35]. This study found that ROS induced by Cd stress significantly inhibited the protein expression of psb and psa family genes in photosystem II and photosystem I, the structural stability of PSII and PSI, light energy capture, and electron transfer processes. This interferes with the functional roles of PSII and PSI in rice seedlings [3, 36]. This may be because the competition between Cd^2+^ and the bivalent cation (Ca^2+^) in plants destroys the stability of CaMn4 inorganic clusters and inhibits the coding process of the PSB family through excessive accumulation of ROS, thus inhibiting plant photosynthesis [25]. However, compared with the Cd group, the expressions of psb28, psbQ, psbO, psbR, psbP, psaG, psaL, psaD, psaE and psaF functional genes were significantly up-regulated under CdSeL treatment. This suggests that Se supplementation can mitigate the adverse effects of heavy metal stress by preserving the normal function and activity of PSII and PSI [43], thereby enhancing the light utilization efficiency and photosynthetic performance of rice seedlings.

Type ATPase is mainly located on the chloroplast thylakoid membrane and comprises F1 and F0. F1 is located in the cytoplasmic matrix and contains multiple subunits, including three α subunits, three β subunits, one γ subunit, one δ subunit, and one ε subunit. The F1 component is primarily responsible for catalyzing the synthesis of ATP by combining ADP and inorganic phosphoric acid (Pi), thus providing the necessary energy for various cellular processes, including plant carbon metabolism [20]. In this study, it is evident that the expression of atpG and atpH functional genes, which encode components of the F-type ATPase, was significantly down-regulated in rice seedlings under Cd stress. However, when rice seedlings were treated with CdSeL (low-concentration selenium), the expression of these two functional genes was markedly up-regulated compared to the Cd-stressed group. This suggests that the exogenous supplementation of Se has a positive impact on ATP synthesis in rice seedlings, particularly under the conditions of Cd stress. Se supplementation enhances the expression of atpG and atpH genes, which is crucial for F-type ATPase activity. As a result, there is an increased production of ATP in rice seedlings under CdSeL treatment. This elevated ATP synthesis can facilitate the photosynthesis of photosynthetic pigments in rice, thereby improving the photosynthetic capacity and promoting carbon metabolism. The adequate supply of ATP ensures that essential metabolic processes within the plant continue to function normally, contributing to overall plant health and growth [19, 20]. In summary, our study demonstrates that the addition of low concentration of Se can enhance the expression of atpG and atpH functional genes in rice seedlings, leading to increased ATP synthesis. This enhanced ATP production supports the biosynthesis of photosynthetic pigments and various metabolic processes in rice, particularly when plants are exposed to the stress of Cd.

The PetH and PetF genes play a significant role in photosynthesis, specifically in maintaining the redox balance of glutathione (GSH/GSSG) in plants. These genes encode ferredoxin NADP + reductase, an enzyme responsible for the conversion of NADP + to NADPH during the final stages of the photosynthetic electron transport chain. NADPH is crucial for various metabolic processes in plants and is particularly important for maintaining the balance of oxidized and reduced glutathione, which is essential for managing oxidative stress [24]. Our study indicates that under Cd stress, the expression of PetH and PetF functional genes (Fig. 5) in rice seedlings is upregulated. This suggests that when rice plants are exposed to Cd-induced oxidative stress, they respond by increasing the expression of these genes. This response likely helps in the rapid clearance of reactive oxygen species (ROS) generated due to Cd stress, thus ensuring the normal functioning of chloroplasts and other cellular processes. Interestingly, when rice seedlings were treated with low-concentration selenium (CdSeL), the expression of PetH and PetF genes was further enhanced. This observation suggests that selenium supplementation can potentiate the response to Cd stress. Previous research, such as the study by Jiao et al., has also demonstrated that under high concentrations of Cd stress, the expression of these genes in plants (e.g., wheat) can be significantly up-regulated [19]. However, the addition of exogenous Se, particularly at low concentrations, can further increase the expression levels of PetH and PetF genes, potentially enhancing the plant's ability to manage oxidative stress induced by Cd. It's important to note that the response to selenium can be concentration-dependent, as our study suggests. While low concentrations of Se seem to enhance the expression of these genes and promote the GSH-GSSG cycle, high selenium concentrations may lead to different responses, such as decreased expression of these genes. Therefore, maintaining an optimal selenium concentration is crucial for maximizing its potential benefits in mitigating the effects of Cd stress on plants and promoting their tolerance to such stressors.

The LHCA and LHCB functional gene families play a crucial role in photosynthesis, encoding the formation of chlorophyll protein complexes (LHC) to capture photons, thereby stimulating the plant's photosynthetic system to function. The LHCB complex is mainly composed of four subunits: LHCB1, LHCB2, LHCB3, and LHCB6, and the LHCA complex is mainly composed of four subunits: LHCA1, LHCA2, LHCA3, and LHCA4 [1, 8]. These subunits contain chlorophyll molecules that can encode chlorophyll a/b binding proteins in photosystem II. This encoding extends the absorption spectrum of light energy, thereby enhancing the efficiency of light energy utilization and ultimately improving photosynthesis efficiency. This process involves absorbing a broader range of light energy, subsequently converting it into electron energy. Consequently, this promotes electron transport, ATP synthesis, and NADPH production within the photosynthetic process, as elucidated in the previous study [25, 30]. In this study, it was found that the expressions of LHCA and LHCB functional gene families in rice seedlings were significantly down-regulated under heavy metal Cd stress, which was consistent with the down-regulated expression of LHCA and LHCB functional gene families in rice discovered by Wang et al. under Cd stress [45]. However, under the treatment of CdSeL, the functional gene families of LHCA and LHCB were significantly upregulated in rice seedlings compared with the Cd group. Studies have shown that exogenous application of Se can reduce the toxic effect of Cd on rice and directly affect rice's photosynthetic rate, thus promoting plants' biomass assimilation [12]. Therefore, the addition of low concentration Se can significantly promote the light energy utilization efficiency of rice seedlings under Cd stress and improve the photosynthetic efficiency to promote rice's growth and development under Cd stress.

Our study, along with several others, provides valuable insights into the beneficial effects of Se in mitigating the toxic effects of Cd in rice. Se has been shown to enhance plant stress resistance and promote growth and development, particularly under conditions of heavy metal stress like Cd [13, 14]. Transcriptome analysis conducted in our study (Figs. 1, 2, 3, 4 and 5) revealed that Se-regulated DEGs are involved in various pathways critical for plant functioning under Cd stress. These pathways include those related to the electron transport chain, photosynthesis, photosynthetic electron transport in photosystem I, light harvesting photosystem I, tetrapyrrole biosynthetic process, NAD(P)H dehydrogenase complex assembly, porphyrin-containing compound biosynthetic process, and photosystem II oxygen-evolving complex. These findings suggest that Se broadly impacts multiple aspects of plant physiology and metabolism to counteract the adverse effects of Cd stress. Furthermore, our study employed weighted gene co-expression network analysis (WGCNA) to identify hub genes in specific modules. The hub genes were found to be primarily associated with processes related to photosynthetic pigment biosynthesis, photosystem stability regulation, photon capture, electron transfer, energy supply, and ROS clearance. These results provide evidence of the complex regulatory mechanisms by which Se enhances Cd-stressed plants' ability to maintain photosynthesis and overall vitality. Proteomic analysis in our study corroborated the transcriptomic findings, highlighting that Se mediates processes involved in porphyrin metabolism, carbon fixation in photosynthetic organisms, biosynthesis of secondary metabolites, glyoxylate and dicarboxylate metabolism, carbon metabolism, carotenoid biosynthesis, pentose phosphate pathway, photosynthesis and ribosome function (Figs. 7 and 8). These findings suggest that Se supplementation positively influences key metabolic pathways, reinforcing the importance of Se in facilitating the biosynthesis of photosynthetic pigments and metabolites that are essential for plant growth and stress tolerance. The study also underscores the role of Se in enhancing plants' antioxidant stress systems. Se at low concentrations has been demonstrated to induce and strengthen antioxidant defenses in plants, including the activities of antioxidant enzymes POD, SOD, APX, and CAT, and increase the contents of non-enzymatic antioxidant substances GSH, AsA, flavonoids, and PRO, to clear the excessive ROS induced and accumulated by heavy metal stress and improve the resistance of plants to abiotic stress. They enhanced plants' antioxidant properties [2, 21, 39]. In summary, our study provides a comprehensive understanding of the multi-faceted mechanisms through which selenium can improve rice plant tolerance to cadmium stress. This includes its effects on metabolic pathways, photosynthesis, antioxidant defenses, and overall growth and development. These insights have implications for strategies to mitigate the adverse effects of heavy metal contamination on crop plants and enhance their productivity in polluted environments.

In our study, Se can enhance the expression of functional genes and proteins in photosynthesis, thereby promoting its energy supply under heavy metal stress and thus enhancing the dynamic response process of its resistance mechanism. The advantages of Selenium-rich fertilizers over other biological and non-biological fortification technologies include: (1) selenium can change soil pH and control the mobility of metal ions; (2) Selenium promotes the absorption of trace elements in plant roots and enhance the expression of plant functional pathways. (3) Se could restore some of the impaired physiological and metabolic processes in response to stress, thereby alleviating the toxicity of trace elements and improving the tolerance of plants; (4) Selenium promotes the formation and retention of metal chelates in vacuoles; (5) Selenium promoted the formation of iron film and requested some metal elements on the root surface. (6) Selenium alters the subcellular distribution of elements into specific organelles and cellular sites; (7) Selenium regulates functional gene and protein expression, increases pectin and hemicellulose content in plants, thickens cell walls, and reduces the transfer of elements between cell membranes 11,33,59. Our previous work using arbuscular mycorrhizal fungi to enhance plant tolerance to heavy metals has shown that colonizing beneficial microbial communities in plant rhizosphere can effectively promote plant photosynthesis and metabolic processes. However, applying microbial remediation technology is complex, mainly because the microorganisms are too tiny to collect and control. And subsequent recycling and processing work. In addition, the metabolic activity of microorganisms is greatly affected by environmental factors, such as soil nutrient conditions, temperature, and moisture, and environmental differences can cause instability in the restoration effect. Furthermore, the more significant variability of microorganisms may alter their intended transformation pathways to soil heavy metal pollutants and produce more toxic derivatives that potentially harm the soil environment 47,60. Therefore, improving plant growth mediated by Se is more advantageous in practical applications.

### The addition of exogenous Se alleviates oxidative damage caused by Cd stress

Throughout long-term evolutionary processes, plants have evolved defense mechanisms to combat the toxic effects of Cd stress. Among these mechanisms, both enzyme-driven and non-enzyme-driven antioxidant systems play a crucial role in coping with oxidative stress. These systems involve processes like the AsA-GSH cycle, GSH-GSSG cycle, SOD, POD, and CAT enzyme activity [7, 46]. This study found that under Cd stress, the contents of malondialdehyde (MDA), protein carbonyl groups, and hydroxyl radicals (·OH) in rice seedlings increased significantly. This elevation in oxidative markers intensified membrane lipid peroxidation, causing oxidative damage to the plants (Fig. 1D). Moreover, transcriptome and proteome analyses showed that multiple pathways involved in DEGs and DEPs directly influenced the processes related to peroxisome function, peroxisomal processes, and energy metabolism (Figs. 6, 7 and 8). Under normal conditions, plants maintain a dynamic equilibrium of ROS, using them as signaling molecules rather than causing oxidative damage. ROS are primarily involved in regulating signal transduction processes and biochemical responses within plants. These include the transmission of oxidative stress signals between organelles, intercellular signaling, Ca^2+^ signal transduction, and mitogen-activated protein kinase (MAPK) signal transduction pathways [20, 27]. Notably, this study found that in the comparison group of CK vs Cd and Cd vs CdSeL, DEGs were generally observed to participate in the signal transduction process of plants. MAPK signaling pathway—Plant and Plant hormone signal transduction significantly enriched the first 20 KEGG pathways (Figs. 4 and 5). The results showed that the addition of exogenous Se mediated the hormone signal transduction pathway in rice seedlings and alleviated the toxic effect of Cd stress on rice seedlings.

Previous studies have shown that applying selenate can significantly up-regulate the expression of functional genes encoding IAA, ABF, and JAZ, regulate plant hormone signal transduction pathways, and thus enhance the tolerance of rice to Cd stress [17]. At the same time, the functional genes (PYL, EIN3, ERF1, SNRK2, NME, IAA, ABF, and JAZ) involved in encoding plant hormones in rice seedlings did not change significantly under Cd stress. However, under CdSeL treatment, the coding functional genes of all MAPK signal transduction pathways and most plant hormone signal transduction coding functional genes showed a significant up-regulation trend (Fig. 5). The functional genes encoding the MAPK and plant hormone signal transduction pathways in plants can cooperate and interact to activate their transcription factors for expression. Thus, photosystem I (PsaN, PsaE, OsPS1, PsaK, PsaD, and PSAH), photosystem II (PSBS1, OsPsbR3, PsbR, and PsbO), REDOX enzymes (OsFdC1, Fd1, and OSFDC1) are activated OsLFNR1), photosynthetic pigments (PDS1, OsABA1, CYP97A4, and OsLCYe), photon capture (ASCAB9, CabE, CAB2R, and LHCB), Carbon metabolism (Osppc, RCS3, AIM1, PGK, PEPC, Aldo, CLA1, GADPH, GLO4, ALT, CATC, RBCS, and FR) and Glutathione metabolism (OsGSTF1, OsGSTF2, OsGSTU35, OsGSTU11, OSG6PDH1, OsGSTU3, OsG6PDH2, OsG6PDH4, OsGSTU13, OsGpx4, OsDHAR1, OsAPx7, APX8 and Functional protein expression of OsPrxII (Fig. 8), heavy metal ion transport and energy metabolism (OsMYB60, PRX131, OsCDGSH, HXK7, and OscytME2) (Fig. 3), and gene expression of the antioxidant stress system (CAT, GSH, flavonoid, and GSH-GSSG cycles) (Figs. 5 and 6) Thus, the toxic damage to rice induced by Cd stress was alleviated, and rice growth was promoted (Figs. 1 and 2).

### The addition of exogenous Se regulated the cell wall metabolism of rice seedlings under Cd stress

The cell wall constitutes a major component of plant cell structure, primarily composed of cellulose (hemicellulose-1 and hemicellulose-2), along with a substantial presence of matrix polysaccharides (glucans, heteromannans, and pectin polysaccharides). Additionally, it contains other substances (such as lignin) [5, 29]. These cell wall constituents feature molecular structures rich in functional group structures (-NH_2_, -COOH, -SH, and -OH), which can effectively bind heavy metal ions, reducing their bioavailability and alleviating their toxic effects on plants [22, 55]. This study showed that following CdSeL treatment, there was a significant increase in the levels of hemicellulose and pectin in rice seedlings. Moreover, the functional genes encoding heavy metal ion binding (C4H1, PRX family, and atp6), cell wall metabolism (OsPAL07, 4CL5, OsCAD3, and C4H2), and oxidative detoxification (Pox1) in the phenylpropanoid biosynthesis pathway showed a significantly up-regulated trend (Fig. 5E). Previous research has demonstrated that the activation of the phenylpropanoid biosynthesis pathway can lead to the accumulation of various secondary metabolites in plant tissues, including lignin, isoflavones, terpenes, cinnamic acid, ferulic acid, Casparian strips, suberin and glycerol [37, 53, 56]. However, the role of Se-mediated gene regulation in the rice phenylpropanoid biosynthesis pathway under Cd stress remains to be fully understood. It is worth noting that studies by Jia et al. have demonstrated that pectin in the cell wall contains numerous negatively charged groups, facilitating the binding of heavy metal ions and significantly enhancing the cell wall's ability to immobilize these ions [18]. Additionally, metal ions can form complexes with hemicellulose, thus mitigating their toxicity [26, 32]. Furthermore, the Casparian strips and suberin layers serve as critical defense barriers in plants, effectively sequestering heavy metal ions and restricting their entry into the plant cells. The antioxidant properties of compounds like cinnamic acid, ferulic acid, and flavonoids can remove excessive free radicals in plants, thus enhancing the resistance of plants to heavy metal stress [25]. In summary, the biosynthesis of phenylpropanoid compounds within the cell wall metabolism may represent a potential mechanism by which Se mediates the alleviation of Cd toxicity in rice plants.

## Conclusions

**Fig. 10:**
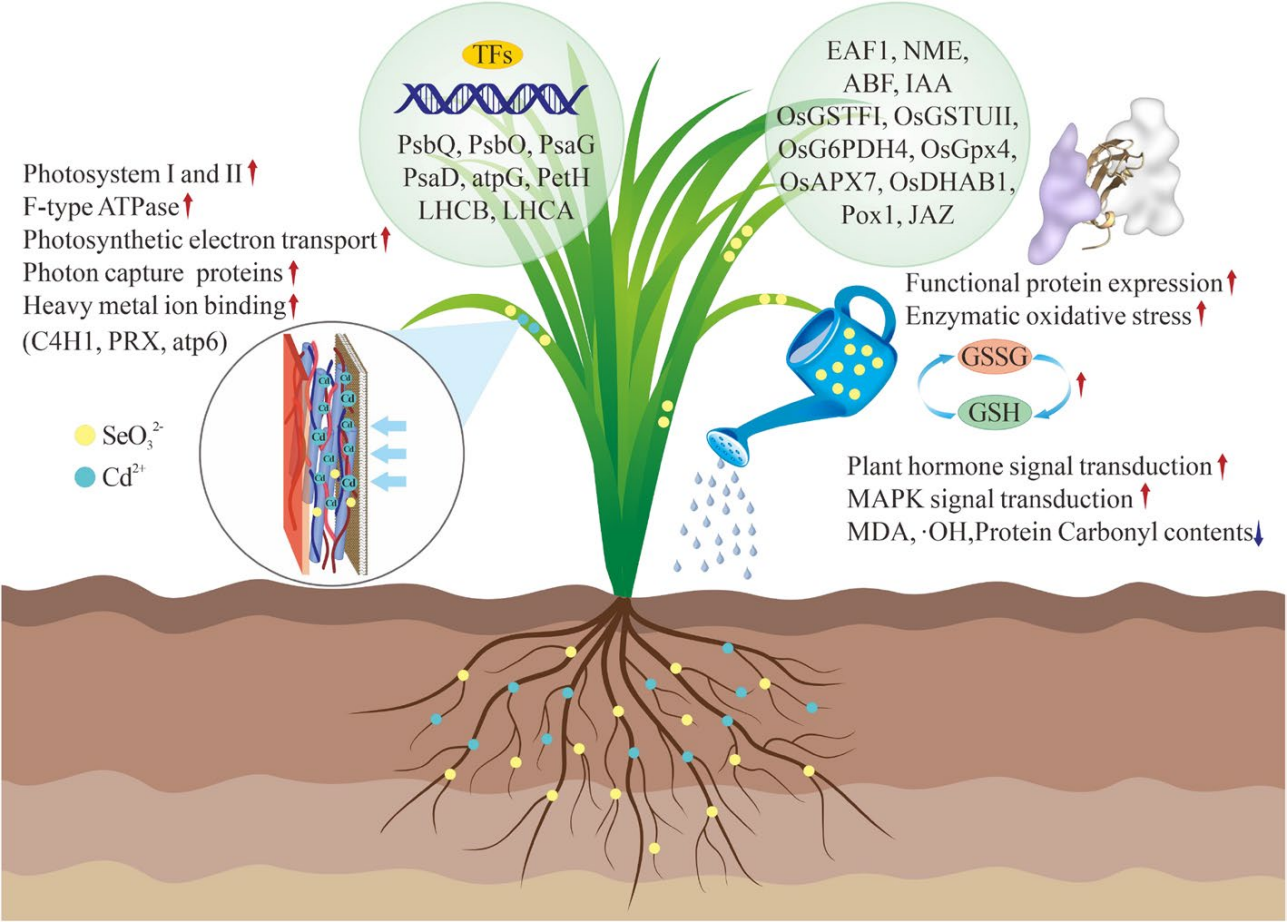
Schematic diagram of potential mechanisms of Se-mediated mitigation of Cd toxicity in rice seedlings

The toxic effect of Cd stress on rice seedlings significantly inhibited plants' daily metabolic function. However, adding exogenous selenium effectively improved the effect of cadmium stress on plants. Se restored the REDOX homeostasis of rice seedlings by regulating the activities of antioxidant enzymes (CAT, POD, and SOD) and the contents of non-enzymatic antioxidants (GSH, AsA, PRO, Cys, and flavonoids), as well as the GSH-GSSG and GSH-ASA cycling processes, and reduced the oxidative damage of reactive oxygen species in rice seedlings. At the same time, the expression of functional genes psbQ, psbO, psaG, psaD, atpG, PetH, LHCA, LHCB family, and C4H1, PRX, atp6 were up-regulated, which enhanced the energy metabolism process and photon capture ability of photosystem I and photosystem II in photosynthesis of rice seedlings. Moreover, it enhances the binding ability of plants to heavy metal ions. It mediates the up-regulated expression of functional proteins OsGSTF1, OsGSTU11, OsG6PDH4, OsDHAB1, CP29, and CabE and promotes stress regulation of photosynthesis and antioxidant systems in rice seedlings. At the same time, it regulates the plant hormone signal transduction pathway and MAPK signal pathway, up-regulates the expression response process of IAA, ABA, and JAZ, activates the synergistic effect between each cell, and rapidly maintains the homeostasis and REDOX homeostasis of plants (Fig. 10). Therefore, adding exogenous Se alleviated the toxic effects of Cd stress on rice and promoted rice growth. The findings in this study provide a valuable theoretical basis for understanding the molecular response mechanisms of Se-mediated cadmium resistance in rice. It also provided technical support for crop production safety in cadmium-contaminated soil.

### Supplementary Information

**Supplementary Material 1.**

**Supplementary Material 2.**

**Supplementary Material 3.**

**Supplementary Material 4.**

## Data Availability

The data that support the findings of this study are available from [Shanghai MajorbioBio-pharm Biotechnology Co., Ltd. (Shanghai, China)] but restrictions apply to the availability of these data, which were used under license for the current study, and are not publicly available. Data are available from the authors upon reasonable request, and with permission of [Shanghai MajorbioBio-pharm Biotechnology Co., Ltd. (Shanghai, China)].
