# Supplementary material for: Utilizing transcriptomics and proteomics to unravel key genes and proteins of Oryza sativa seedlings mediated by selenium in response to cadmium stress
Source: BMC Plant Biol. 2024 May 3;24:360. doi: 10.1186/s12870-024-05076-7 (PMC11067083; doi:10.1186/s12870-024-05076-7)
Supplement: Supplementary file 1 — Supplementary Material 1. [file 12870_2024_5076_MOESM1_ESM.docx]

**Table S1** Evaluation statistics of sequencing data of underground samples of Oryza sativa. CK, control; Cd, 3 mg/kg Cd; CdSeL, 3 mg/kg Cd+1 mg/kg Se; CdSeH, 3 mg/kg Cd+5 mg/kg Se.

| **Treatment** | **Sample** | **Raw reads** | **Raw bases** | **Clean reads** | **Clean bases** | **Error rate(%)** | **Q20(%)** | **Q30(%)** | **GC content(%)** |
| --- | --- | --- | --- | --- | --- | --- | --- | --- | --- |
| CK | CK-1 | 43922652 | 6632320452 | 42828806 | 6199220665 | 0.0243 | 98.27 | 95 | 48.18 |
|  | CK-2 | 41842200 | 6318172200 | 41145178 | 6073139173 | 0.0242 | 98.32 | 95.03 | 48.44 |
|  | CK-3 | 44030572 | 6648616372 | 43546176 | 6503536790 | 0.0242 | 98.34 | 95.02 | 48.52 |
| Cd | Cd-1 | 41594204 | 6280724804 | 40978004 | 6037223771 | 0.024 | 98.39 | 95.21 | 47.37 |
|  | Cd-1 | 44594778 | 6733811478 | 43970378 | 6540664295 | 0.0241 | 98.38 | 95.16 | 48.46 |
|  | Cd-1 | 41937804 | 6332608404 | 41414722 | 6177055946 | 0.0244 | 98.26 | 94.86 | 48.76 |
| CdSeL | CdSeL-1 | 43200502 | 6523275802 | 42722214 | 6330114634 | 0.0242 | 98.33 | 95.08 | 48.09 |
|  | CdSeL-2 | 50417080 | 7612979080 | 49533330 | 7257800959 | 0.0241 | 98.34 | 95.13 | 48.91 |
|  | CdSeL-3 | 44950498 | 6787525198 | 43971334 | 6392591644 | 0.0245 | 98.2 | 94.81 | 49.2 |
| CdSeH | CdSeH-1 | 45584372 | 6883240172 | 44640060 | 6496310341 | 0.0242 | 98.3 | 95.02 | 47.77 |
|  | CdSeH-2 | 45221650 | 6828469150 | 44510676 | 6502579124 | 0.0239 | 98.49 | 95.38 | 46.7 |
|  | CdSeH-3 | 46143840 | 6967719840 | 45401740 | 6655430031 | 0.0243 | 98.3 | 94.97 | 46.43 |

**Table S2** Statistics of up-regulated and down-regulated DEGs. CK, control; Cd, 3 mg/kg Cd; CdSeL, 3 mg/kg Cd+1 mg/kg Se; CdSeH, 3 mg/kg Cd+5 mg/kg Se.

| **group** | **total** | **up** | **down** |
| --- | --- | --- | --- |
| Cd_vs_CK | 533 | 263 | 270 |
| CdSeL_vs_Cd | 2809 | 1996 | 813 |
| CdSeH_vs_Cd | 1325 | 488 | 837 |
| CdSeL_vs_CK | 2251 | 1688 | 563 |
| CdSeH_vs_CK | 1100 | 411 | 689 |

**Table S3.** List of primers used for qRT-PCR

|  | | |
| --- | --- | --- |
| **Gene/Locus** | **Forward Primer** | **Reverse Primer** |
| LOC_Os08g36480 | GCCAACTCGTCGACAAGAAG | TGATGAGGAGGTCGAAGTGG |
| LOC_Os08g36920 | GACGAGCAAGTAGAGCACAC | AGAATCTGACGACTGTGGGA |
| LOC_Os07g34070 | AGTCCTTGAACCACCACCAG | TCGTGTTCATCAGCTTCGAC |
| LOC_Os01g55240 | GCGACTCCTTCTTCGTCAAC | TGCAATCCTCTGTGCTAACG |
| LOC_Os07g05940 | CAGATCGTGTTCAAGCTCCA | AGAGGTGGAAGCAGAAGCAG |
| LOC_Os09g20390 | GGGAAGCAGATCGTCATGTT | TCACGAACTCGAACACCTTG |
| LOC_Os06g04590 | ATGGCGATCAAGAAGGGCG | ACTCGCTGACCTGCTTCTG |
| LOC_Os03g13140 | CGTGGAGGATAACAATGCCG | AGGAACGAGAACATCTGGCT |
| LOC_Os02g53130 | ACAGCATCATGATCAACGCC | TAGGGCTCTCAACGATGGAC |
| LOC_Os02g48360 | GATGAAATGAACAGGCGTATGA | TAGGCGTAATCGCAGTCAAA |
| LOC_Os12g08090 | CAGCTCCAGCAACCCTTACA | ATTGTCTGGACGATGCCGAG |
| LOC_Os09g35010 | GGCGACGAAGAAGAAGACAA | AGTAGCTCCAGAGCGGCATG |
