# Supplementary material for: Utilizing transcriptomics and proteomics to unravel key genes and proteins of Oryza sativa seedlings mediated by selenium in response to cadmium stress
Source: BMC Plant Biol. 2024 May 3;24:360. doi: 10.1186/s12870-024-05076-7 (PMC11067083; doi:10.1186/s12870-024-05076-7)
Supplement: Supplementary file 4 — Supplementary Material 4. [file 12870_2024_5076_MOESM4_ESM.pdf]

Fig. S1 Identification of differentially expressed genes (DEGs) in comparison groups. (A) DEGs Veen diagram, (B) DEGs PCA analysis, (C) Bar graph showed the upregulated and downregulated DEGs of different comparison groups in different colors, upregulated (red) and downregulated (blue). (CK: control, Cd: 3 mg/kg Cd, CdSeL: 3 mg/kg Cd + Se 1 mg/kg, CdSeH: 3 mg/kg Cd + Se 5 mg/kg).

Fig. S2 The top 20 GO term annotation analysis of all annotated DEGs in CK vs Cd (A), CdSeL vs Cd (B), CdSeH vs Cd (C), CdSeH vs CK (D), CdSeL vs CK for leaf. The Y-axis represented the GO term; the X-axis represented the annotated number of DEGs. (CK: control, Cd: 3 mg/kg Cd, CdSeL: 3 mg/kg Cd + Se 1 mg/kg, CdSeH: 3 mg/kg Cd + Se 5 mg/kg).

Fig. S3 The top 20 KEGG term annotation analysis of all annotated DEGs in CK vs Cd (A), CdSeL vs Cd (B), CdSeH vs Cd (C), CdSeH vs CK (D), CdSeL vs CK for leaf. The Y-axis represented the GO term; the X-axis represented the annotated number of DEGs. (CK: control, Cd: 3 mg/kg Cd, CdSeL: 3 mg/kg Cd + Se 1 mg/kg, CdSeH: 3 mg/kg Cd + Se 5 mg/kg).

Fig. S4 Quality control of transcriptome data and Changes in DEGs expression. Heatmap showing the results of the clustering analysis of DEGs. CK vs Cd (A), CdSeL vs Cd (B), CdSeH vs Cd (C). (CK: control, Cd: 3 mg/kg Cd, CdSeL: 3 mg/kg Cd + Se 1 mg/kg, CdSeH: 3 mg/kg Cd + Se 5 mg/kg).

Fig. S5 The top 20 GO term and KEGG pathway enrichment analysis of the key module of WGCNA. The Y-axis represented the GO term and KEGG pathway; the X-axis represented the rich factor of DEGs (Physiological cell wall energy photosynthesis). (CK: control, Cd: 3 mg/kg Cd, CdSeL: 3 mg/kg Cd + Se 1 mg/kg, CdSeH: 3 mg/kg Cd + Se 5 mg/kg).

Fig. S6 The top 20 GO term and KEGG pathway enrichment analysis of the key module of WGCNA. The Y-axis represented the GO term and KEGG pathway; the X-axis represented the rich factor of DEGs (Enzymatic and non-enzymatic antioxidant mechanism). (CK: control, Cd: 3 mg/kg Cd, CdSeL: 3 mg/kg Cd + Se 1 mg/kg, CdSeH: 3 mg/kg Cd + Se 5 mg/kg).

Fig. S7 The top 20 GO and KEGG term annotation analysis of all annotated DEPs in CK vs Cd, CdSeL vs Cd, CdSeH vs Cd for leaf. The Y-axis represented the GO and KEGG terms; the X-axis represented the annotated number of DEPs. (CK: control, Cd: 3 mg/kg Cd, CdSeL: 3 mg/kg Cd + Se 1 mg/kg, CdSeH: 3 mg/kg Cd + Se 5 mg/kg).

Fig. S1

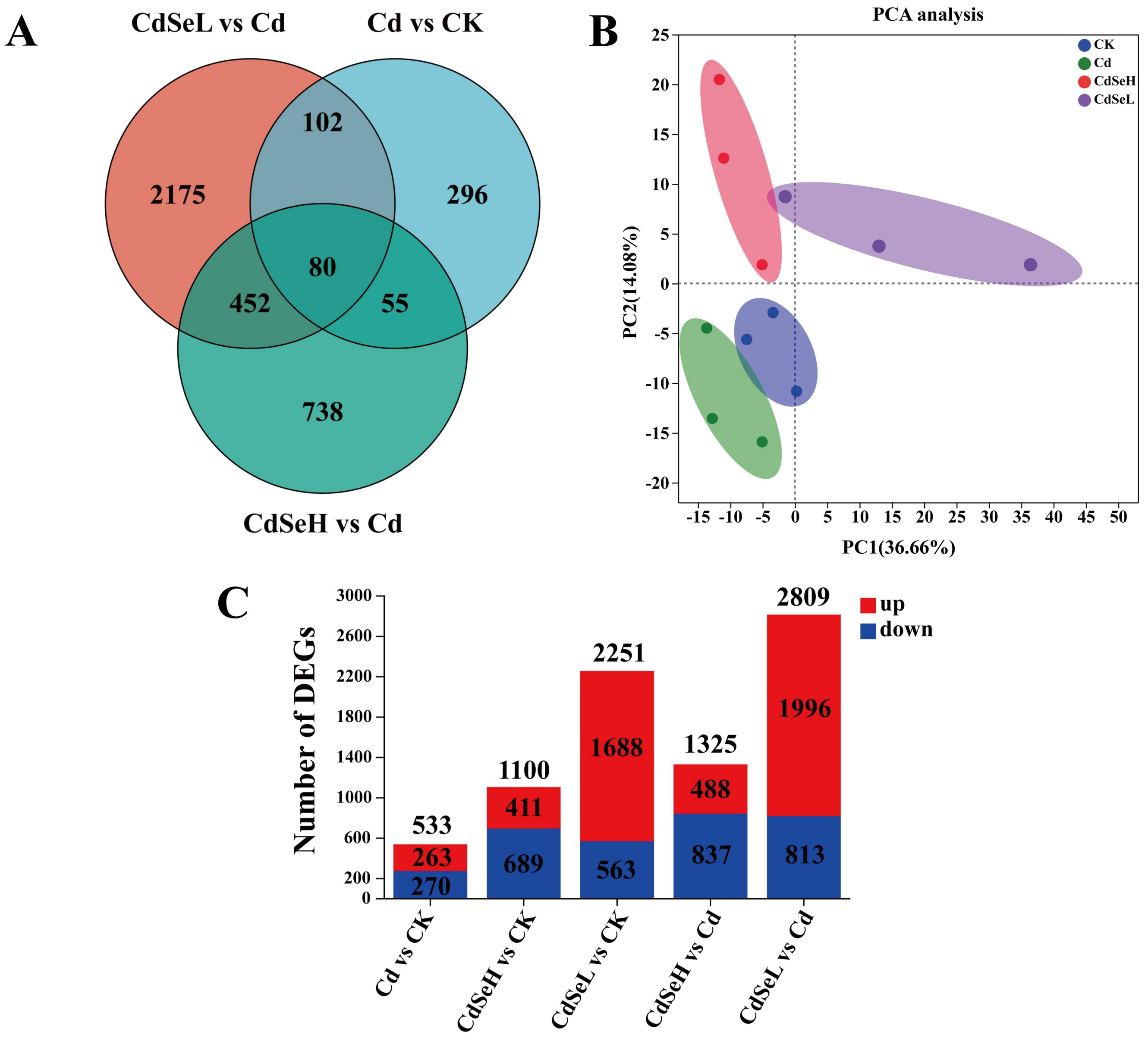
A
CdSeL vs Cd
Cd vs CK
CdSeH vs Cd
2175
102
296
80
452
55
738
B
PCA analysis
CK
Cd
CdSeH
CdSeL
PC2(14.08%)
PC1(36.66%)
25
20
15
10
5
0
-5
-10
-15
-20
-15 -10 -5 0 5 10 15 20 25 30 35 40 45 50
C
Number of DEGs
3000
2600
2200
1800
1400
1000
600
200
0
up
down
533
263
270
1100
411
689
2251
1688
563
1325
488
837
2809
1996
813
Cd vs CK
CdSeH vs CK
CdSeL vs CK
CdSeH vs Cd
CdSeL vs Cd

Fig. S2

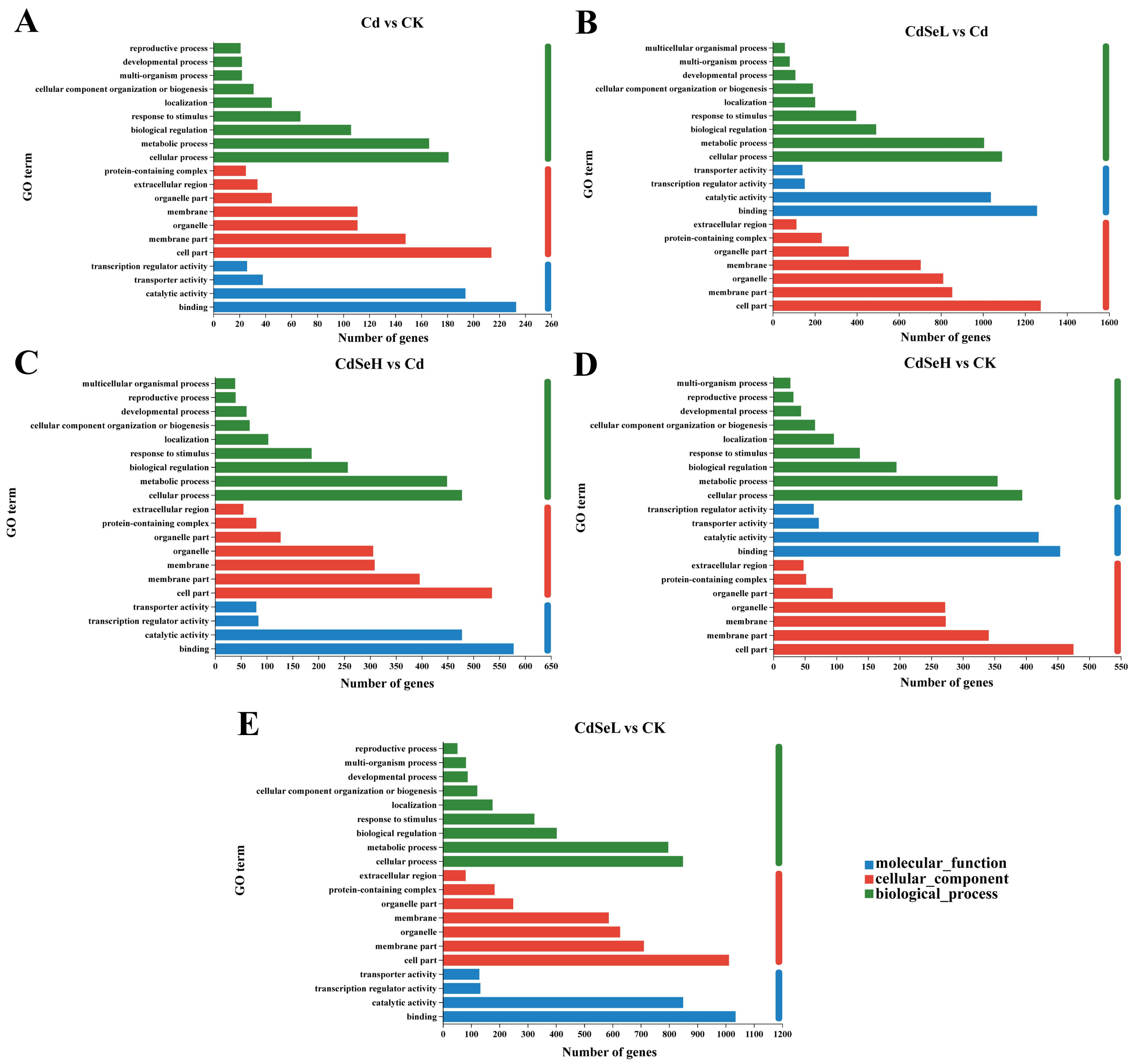
A
Cd vs CK
reproductive process
developmental process
multi-organism process
cellular component organization or biogenesis
localization
response to stimulus
biological regulation
metabolic process
cellular process
protein-containing complex
extracellular region
organelle part
membrane
organelle
membrane part
cell part
transcription regulator activity
transporter activity
catalytic activity
binding
GO term
0 20 40 60 80 100 120 140 160 180 200 220 240 260
Number of genes
B
CdSeL vs Cd
multicellular organismal process
multi-organism process
developmental process
cellular component organization or biogenesis
localization
response to stimulus
biological regulation
metabolic process
cellular process
transporter activity
transcription regulator activity
catalytic activity
binding
extracellular region
protein-containing complex
organelle part
membrane
organelle
membrane part
cell part
GO term
0 200 400 600 800 1000 1200 1400 1600
Number of genes
C
CdSeH vs Cd
multicellular organismal process
reproductive process
developmental process
cellular component organization or biogenesis
localization
response to stimulus
biological regulation
metabolic process
cellular process
extracellular region
protein-containing complex
organelle part
organelle
membrane
membrane part
cell part
transporter activity
transcription regulator activity
catalytic activity
binding
GO term
0 50 100 150 200 250 300 350 400 450 500 550 600 650
Number of genes
D
CdSeH vs CK
multi-organism process
reproductive process
developmental process
cellular component organization or biogenesis
localization
response to stimulus
biological regulation
metabolic process
cellular process
transcription regulator activity
transporter activity
catalytic activity
binding
extracellular region
protein-containing complex
organelle part
organelle
membrane
membrane part
cell part
GO term
0 50 100 150 200 250 300 350 400 450 500 550
Number of genes
E
CdSeL vs CK
reproductive process
multi-organism process
developmental process
cellular component organization or biogenesis
localization
response to stimulus
biological regulation
metabolic process
cellular process
extracellular region
protein-containing complex
organelle part
membrane
organelle
membrane part
cell part
transporter activity
transcription regulator activity
catalytic activity
binding
GO term
0 100 200 300 400 500 600 700 800 900 1000 1100 1200
Number of genes
molecular_function
cellular_component
biological_process

Fig. S3

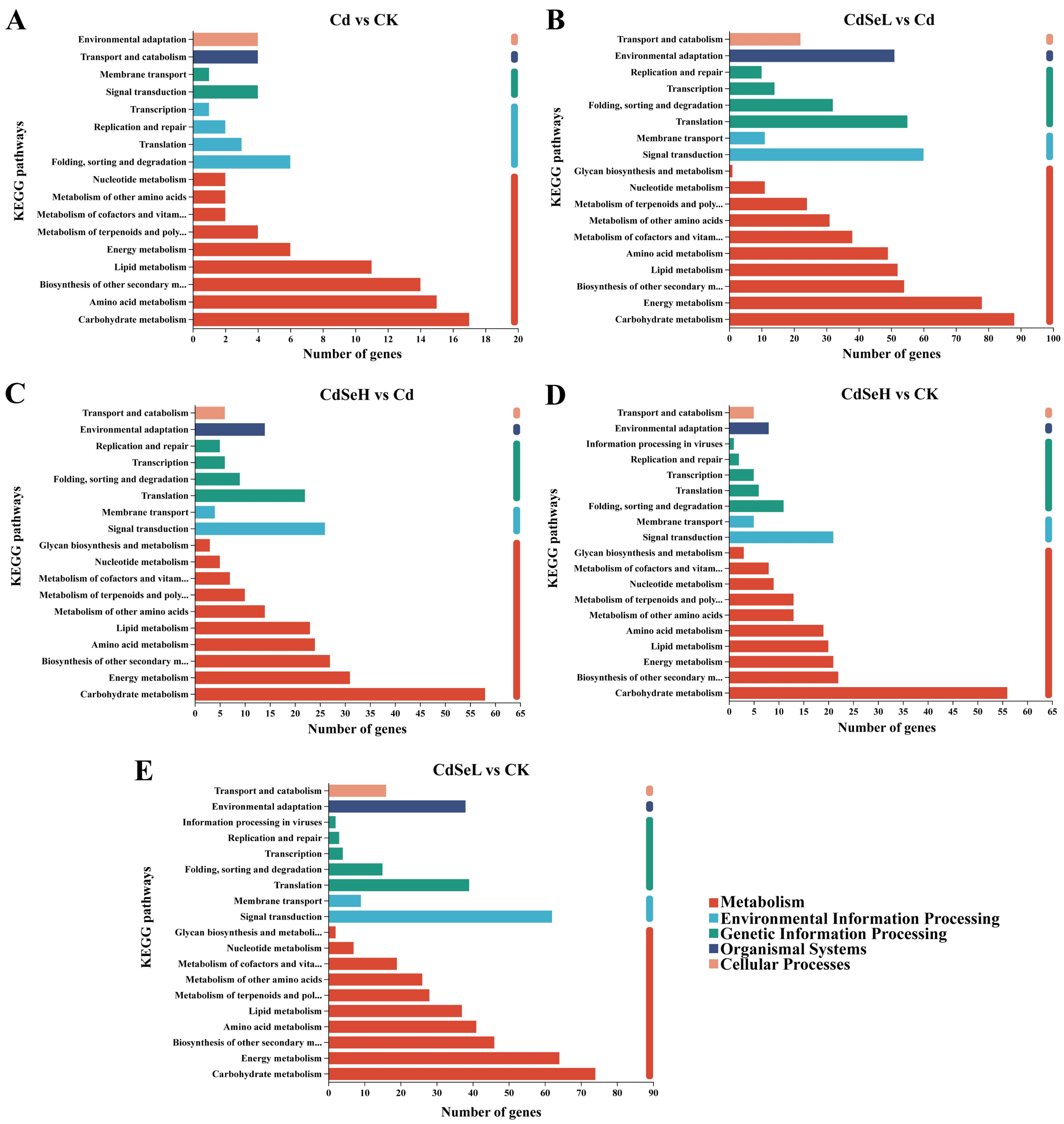
A
Cd vs CK
B
CdSeL vs Cd
C
CdSeH vs Cd
D
CdSeH vs CK
E
CdSeL vs CK
KEGG pathways
Number of genes
Environmental adaptation
Transport and catabolism
Membrane transport
Signal transduction
Transcription
Replication and repair
Translation
Folding, sorting and degradation
Information processing in viruses
Glycan biosynthesis and metabolism
Nucleotide metabolism
Metabolism of other amino acids
Metabolism of cofactors and vitam...
Metabolism of terpenoids and poly...
Energy metabolism
Lipid metabolism
Biosynthesis of other secondary m...
Amino acid metabolism
Carbohydrate metabolism
Metabolism
Environmental Information Processing
Genetic Information Processing
Organismal Systems
Cellular Processes

Fig. S4

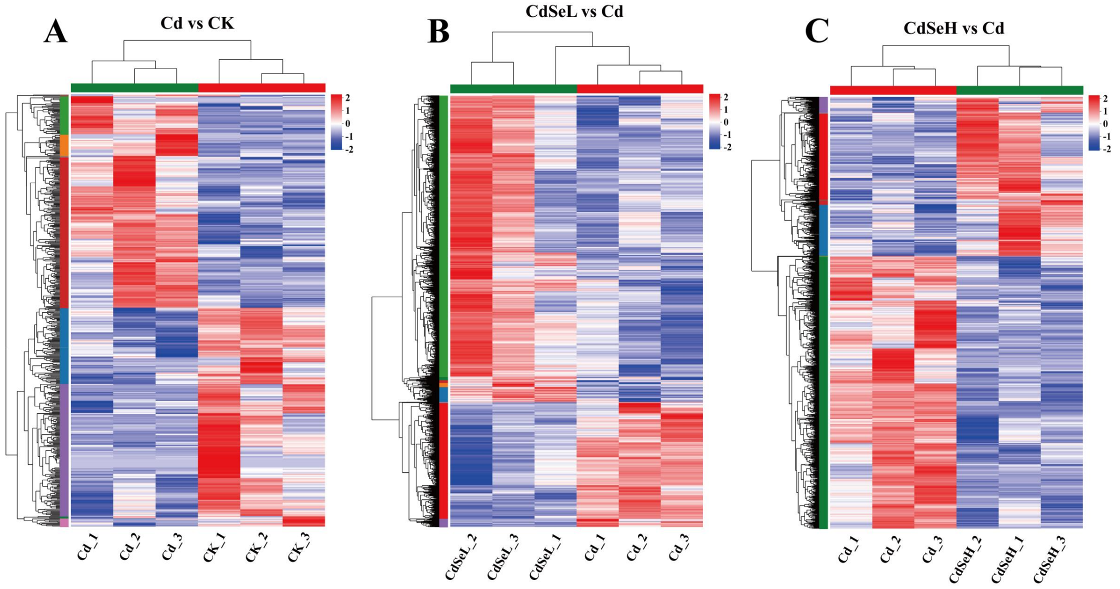
A
Cd vs CK
2
1
0
-1
-2
Cd_1
Cd_2
Cd_3
CK_1
CK_2
CK_3
B
CdSeL vs Cd
CdSeL_2
CdSeL_3
CdSeL_1
Cd_1
Cd_2
Cd_3
C
CdSeH vs Cd
Cd_1
Cd_2
Cd_3
CdSeH_2
CdSeH_1
CdSeH_3

Fig. S5

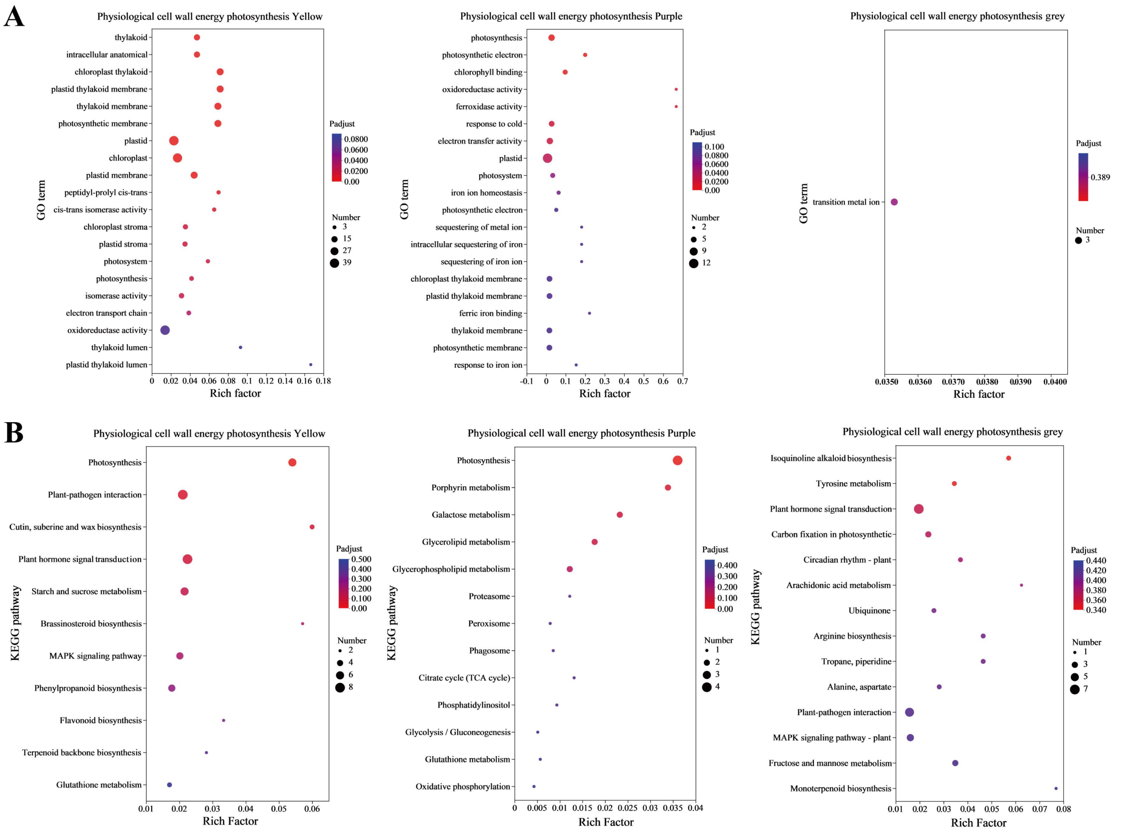
A
Physiological cell wall energy photosynthesis Yellow
thylakoid
intracellular anatomical
chloroplast thylakoid
plastid thylakoid membrane
thylakoid membrane
photosynthetic membrane
plastid
chloroplast
plastid membrane
peptidyl-prolyl cis-trans
cis-trans isomerase activity
chloroplast stroma
plastid stroma
photosystem
photosynthesis
isomerase activity
electron transport chain
oxidoreductase activity
thylakoid lumen
plastid thylakoid lumen
GO term
Rich factor
Padjust
Number
Physiological cell wall energy photosynthesis Purple
photosynthesis
photosynthetic electron
chlorophyll binding
oxidoreductase activity
ferroxidase activity
response to cold
electron transfer activity
plastid
photosystem
iron ion homeostasis
photosynthetic electron
sequestering of metal ion
intracellular sequestering of iron
sequestering of iron ion
chloroplast thylakoid membrane
plastid thylakoid membrane
ferric iron binding
thylakoid membrane
photosynthetic membrane
response to iron ion
GO term
Rich factor
Padjust
Number
Physiological cell wall energy photosynthesis grey
transition metal ion
GO term
Rich factor
Padjust
Number
B
Physiological cell wall energy photosynthesis Yellow
Photosynthesis
Plant-pathogen interaction
Cutin, suberine and wax biosynthesis
Plant hormone signal transduction
Starch and sucrose metabolism
Brassinosteroid biosynthesis
MAPK signaling pathway
Phenylpropanoid biosynthesis
Flavonoid biosynthesis
Terpenoid backbone biosynthesis
Glutathione metabolism
KEGG pathway
Rich Factor
Padjust
Number
Physiological cell wall energy photosynthesis Purple
Photosynthesis
Porphyrin metabolism
Galactose metabolism
Glycerolipid metabolism
Glycerophospholipid metabolism
Proteasome
Peroxisome
Phagosome
Citrate cycle (TCA cycle)
Phosphatidylinositol
Glycolysis / Gluconeogenesis
Glutathione metabolism
Oxidative phosphorylation
KEGG pathway
Rich Factor
Padjust
Number
Physiological cell wall energy photosynthesis grey
Isoquinoline alkaloid biosynthesis
Tyrosine metabolism
Plant hormone signal transduction
Carbon fixation in photosynthetic
Circadian rhythm - plant
Arachidonic acid metabolism
Ubiquinone
Arginine biosynthesis
Tropane, piperidine
Alanine, aspartate
Plant-pathogen interaction
MAPK signaling pathway - plant
Fructose and mannose metabolism
Monoterpenoid biosynthesis
KEGG pathway
Rich Factor
Padjust
Number

Fig. S6

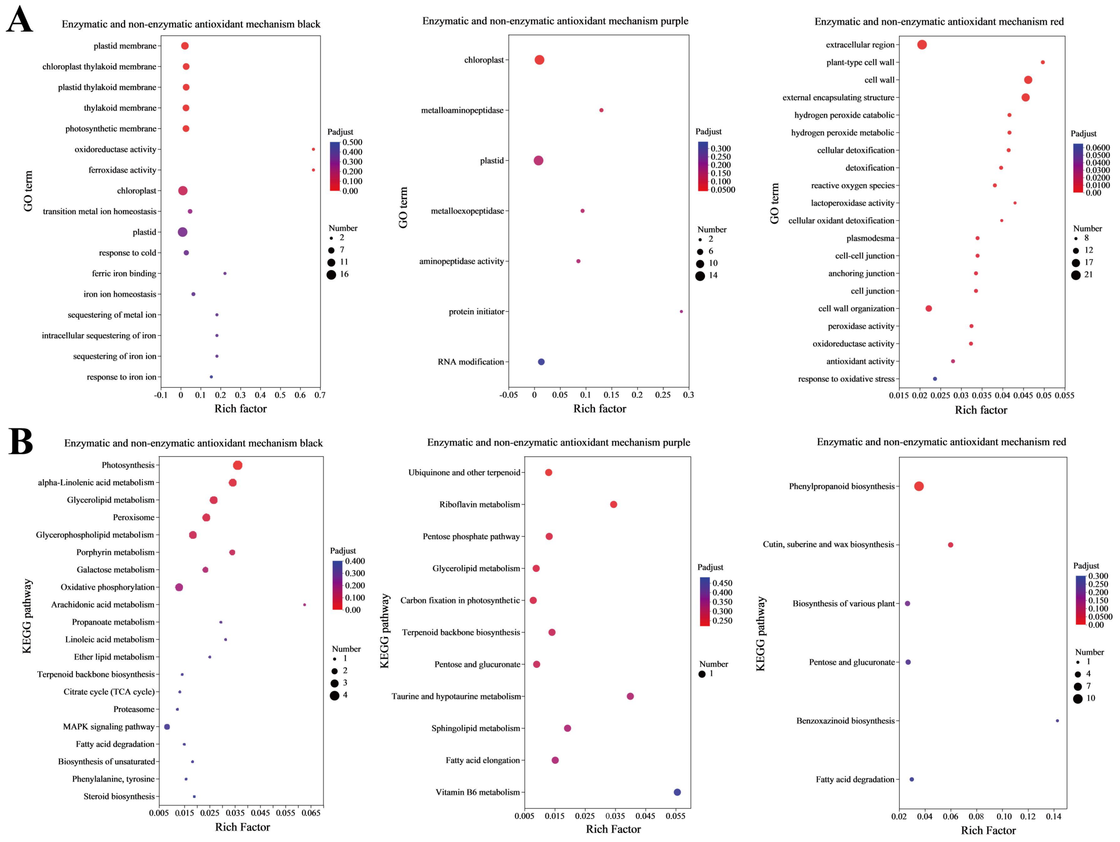

A
Enzymatic and non-enzymatic antioxidant mechanism black
plastid membrane
chloroplast thylakoid membrane
plastid thylakoid membrane
thylakoid membrane
photosynthetic membrane
oxidoreductase activity
ferroxidase activity
chloroplast
transition metal ion homeostasis
plastid
response to cold
ferric iron binding
iron ion homeostasis
sequestering of metal ion
intracellular sequestering of iron
sequestering of iron ion
response to iron ion
GO term
Rich factor
-0.1 0 0.1 0.2 0.3 0.4 0.5 0.6 0.7
Padjust
0.500 0.400 0.300 0.200 0.100 0.00
Number
2 7 11 16
Enzymatic and non-enzymatic antioxidant mechanism purple
chloroplast
metalloaminopeptidase
plastid
metalloexopeptidase
aminopeptidase activity
protein initiator
RNA modification
GO term
Rich factor
-0.05 0 0.05 0.1 0.15 0.2 0.25 0.3
Padjust
0.300 0.250 0.200 0.150 0.100 0.0500
Number
2 6 10 14
Enzymatic and non-enzymatic antioxidant mechanism red
extracellular region
plant-type cell wall
cell wall
external encapsulating structure
hydrogen peroxide catabolic
hydrogen peroxide metabolic
cellular detoxification
detoxification
reactive oxygen species
lactoperoxidase activity
cellular oxidant detoxification
plasmodesma
cell-cell junction
anchoring junction
cell junction
cell wall organization
peroxidase activity
oxidoreductase activity
antioxidant activity
response to oxidative stress
GO term
Rich factor
0.015 0.02 0.025 0.03 0.035 0.04 0.045 0.05 0.055
Padjust
0.0600 0.0500 0.0400 0.0300 0.0200 0.0100 0.00
Number
8 12 17 21
B
Enzymatic and non-enzymatic antioxidant mechanism black
Photosynthesis
alpha-Linolenic acid metabolism
Glycerolipid metabolism
Peroxisome
Glycerophospholipid metabolism
Porphyrin metabolism
Galactose metabolism
Oxidative phosphorylation
Arachidonic acid metabolism
Propanoate metabolism
Linoleic acid metabolism
Ether lipid metabolism
Terpenoid backbone biosynthesis
Citrate cycle (TCA cycle)
Proteasome
MAPK signaling pathway
Fatty acid degradation
Biosynthesis of unsaturated
Phenylalanine, tyrosine
Steroid biosynthesis
KEGG pathway
Rich Factor
0.005 0.015 0.025 0.035 0.045 0.055 0.065
Padjust
0.400 0.300 0.200 0.100 0.00
Number
1 2 3 4
Enzymatic and non-enzymatic antioxidant mechanism purple
Ubiquinone and other terpenoid
Riboflavin metabolism
Pentose phosphate pathway
Glycerolipid metabolism
Carbon fixation in photosynthetic
Terpenoid backbone biosynthesis
Pentose and glucuronate
Taurine and hypotaurine metabolism
Sphingolipid metabolism
Fatty acid elongation
Vitamin B6 metabolism
KEGG pathway
Rich Factor
0.005 0.015 0.025 0.035 0.045 0.055
Padjust
0.450 0.400 0.350 0.300 0.250
Number
1
Enzymatic and non-enzymatic antioxidant mechanism red
Phenylpropanoid biosynthesis
Cutin, suberine and wax biosynthesis
Biosynthesis of various plant
Pentose and glucuronate
Benzoxazinoid biosynthesis
Fatty acid degradation
KEGG pathway
Rich Factor
0.02 0.04 0.06 0.08 0.10 0.12 0.14
Padjust
0.300 0.250 0.200 0.150 0.100 0.0500 0.00
Number
1 4 7 10

Fig. S7

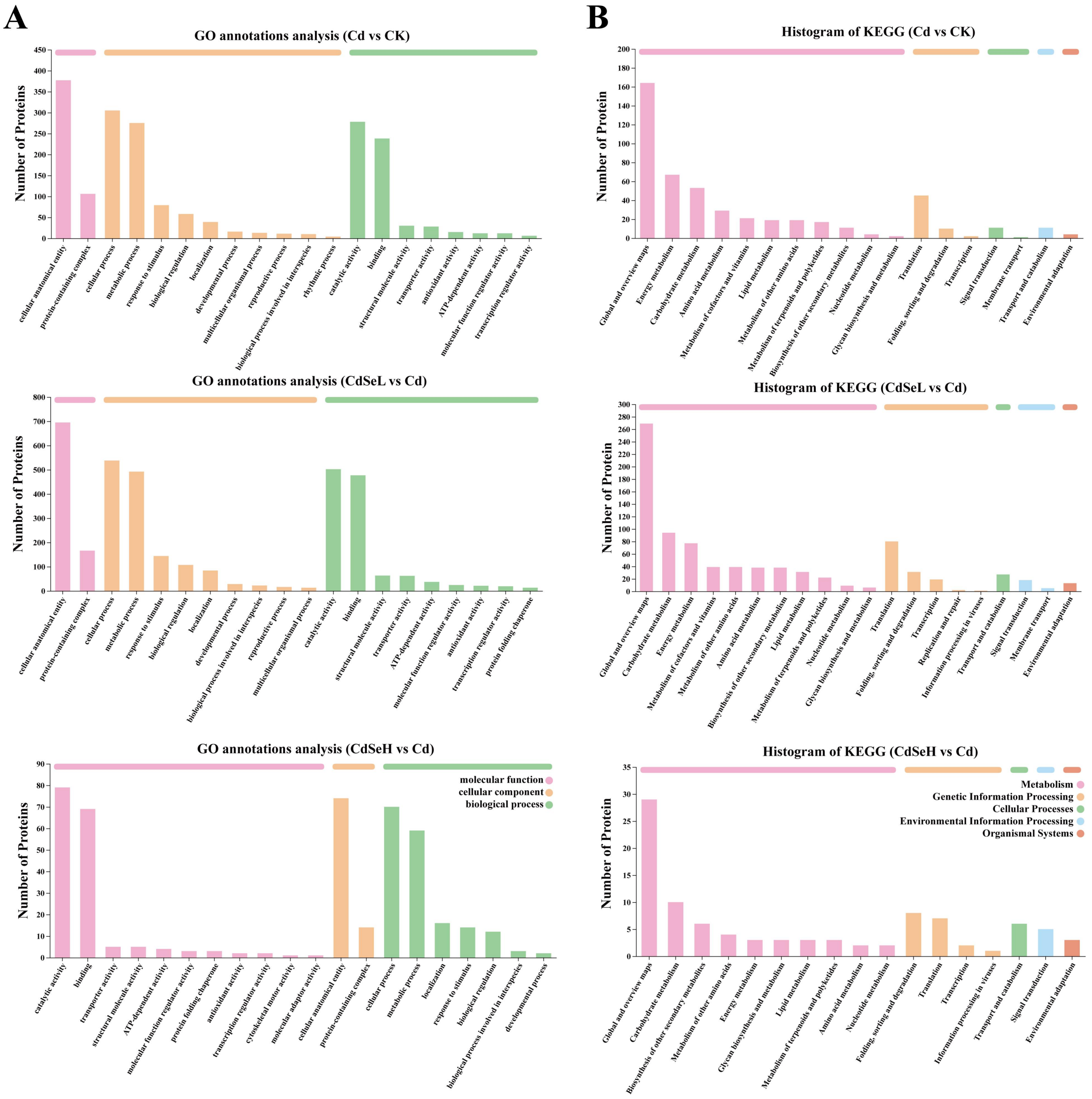
A
B
GO annotations analysis (Cd vs CK)
Histogram of KEGG (Cd vs CK)
GO annotations analysis (CdSeL vs Cd)
Histogram of KEGG (CdSeL vs Cd)
GO annotations analysis (CdSeH vs Cd)
Histogram of KEGG (CdSeH vs Cd)
Number of Proteins
Number of Protein
molecular function
cellular component
biological process
Metabolism
Genetic Information Processing
Cellular Processes
Environmental Information Processing
Organismal Systems
